# A lactylation- and autophagy-associated prognostic signature reveals LSEC-derived CLEC3B as a novel mediator of hepatocellular carcinoma suppression

**DOI:** 10.1371/journal.pcbi.1014426

**Published:** 2026-06-22

**Authors:** Youai Song, Yinkuan Ning, Meihui Li, Jianwei Lan, Liangchen Lei, Yufei Han, Zhuo Meng, Binjie Li, Pengpeng Liu, Quanyan Liu

**Affiliations:** 1 Department of General Surgery, Tianjin Medical University General Hospital, Tianjin, P. R. China; 2 Interventional Vascular Surgery, Shaoyang Central Hospital, Shaoyang, Hunan, P. R. China; Zhejiang University, CHINA

## Abstract

The crosstalk between lactylation and autophagy within the hepatocellular carcinoma (HCC) microenvironment is a burgeoning field with profound implications. By integrating multi-omics data from public cohorts, we delineated two molecular subtypes of HCC with divergent clinical outcomes and established a lactylation-autophagy-related prognostic signature. This signature highlighted CLEC3B as a pivotal gene. Subsequent single-cell RNA sequencing and experimental validation unequivocally pinpointed liver sinusoidal endothelial cells (LSECs) as the principal cellular source of CLEC3B, which was significantly downregulated in HCC tissues. Functionally, conditioned media derived from CLEC3B-overexpressing LSECs potently inhibited HCC cell proliferation. Mechanistic investigations revealed that this tumor-suppressive effect was orchestrated through the concurrent suppression of autophagy and diminution of lactylation levels. Our findings position LSEC-secreted CLEC3B as a novel metabolic mediator in HCC, bridging two key pathways in tumor suppression, and endorse its clinical value both as a prognostic indicator and a promising therapeutic target.

## Author summary

Hepatocellular carcinoma (HCC) is a major global health challenge with limited treatment options and poor prognosis for advanced cases. The tumor microenvironment, particularly the intricate communication between liver cells and surrounding supportive tissues, plays a crucial role in cancer progression. Our study identifies a novel dialogue partner in this environment: liver sinusoidal endothelial cells (LSECs), the specialized lining of liver blood vessels. We discovered that these LSECs secrete a protein called CLEC3B, which acts as a powerful brake on liver tumor growth. Using computational analysis of patient data and laboratory experiments, we show that CLEC3B levels are significantly reduced in liver cancer. When tumor cells are exposed to CLEC3B, their growth is suppressed. We further reveal how CLEC3B works: it simultaneously dampens two key pro-cancer processes inside tumor cells. First, it reduces a specific chemical modification on proteins (driven by lactate, a common byproduct in tumors) known as lactylation. Second, it inhibits autophagy, a cellular recycling process that tumors often hijack for survival. Importantly, our data suggest that the suppression of lactylation is the primary event that leads to the inhibition of autophagy. In summary, our work unveils CLEC3B as a critical tumor-suppressive signal from liver blood vessels. It operates by disrupting a metabolic and self-recycling axis within cancer cells. Restoring CLEC3B signaling or targeting the pathways it controls could represent a promising new strategy for treating liver cancer.

## 1. Introduction

Liver cancer ranks sixth in incidence among all cancers and is the third leading cause of cancer-related deaths worldwide. As the most common type of liver cancer, hepatocellular carcinoma (HCC) accounts for over 80% of all liver cancer cases globally [1,2]. Despite advances in therapeutic strategies, the prognosis for advanced HCC remains poor due to high rates of recurrence, metastasis, and therapy resistance [3]. This clinical predicament underscores the critical need to elucidate the complex molecular mechanisms driving HCC progression. In recent years, research has increasingly shifted towards understanding the pivotal role of the tumor microenvironment (TME), a complex ecosystem where neoplastic cells interact with diverse stromal components, which profoundly influences tumor initiation, progression, and immune evasion [4,5].

Within the liver TME, liver sinusoidal endothelial cells (LSECs) are not merely passive conduits for blood flow but are actively engaged in regulating hepatic physiology and pathology [6–9]. As the most abundant non-parenchymal cells in the liver, LSECs form a unique, fenestrated barrier and are among the first responders to liver injury [8,10,11]. Their dysfunction is a well-established early event in hepatocarcinogenesis. Beyond their structural role, LSECs function as a signaling hub by secreting a plethora of bioactive molecules (the “secretome”) that can directly modulate the behavior of adjacent cells [12]. However, the specific identities and functions of LSEC-derived secreted factors in suppressing or promoting HCC growth remain largely unexplored.

Simultaneously, two pivotal biological processes have emerged as key players in cancer biology: metabolic reprogramming and autophagy. The Warburg effect, a hallmark of cancer metabolism, leads to massive lactate production in the TME. Beyond being a waste product, lactate is now recognized as a key signaling molecule that drives a novel post-translational modification known as lactylation, which regulates gene expression and cellular functions [13–15]. Meanwhile, autophagy plays a context-dependent dual role in HCC, capable of either suppressing tumor initiation or promoting cancer cell survival under stress [16,17].

Intriguingly, the relationship between lactylation and autophagy represents a frontier of significant research potential. Recent pioneering work has begun to elucidate how non-histone lactylation directly governs the autophagic machinery. For instance, lactylation of the transcription factor TFEB has been shown to promote its nuclear translocation and enhance autophagic and lysosomal activity [18]. More recently, a seminal study demonstrated that lactylation of Folliculin (FLCN) at lysine 523 disrupts its interaction with TFEB, thereby suppressing mTORC1 lysosomal localization and promoting TFEB-driven autophagy [19]. Furthermore, beyond transcriptional regulators, matrix stiffness in pancreatic ductal adenocarcinoma has been shown to activate LDHA, driving lactate production that induces lactylation of the transcription factor FOXO3, which in turn sustains persistent autophagy activation [20]. This burgeoning field highlights the significant potential of the lactylation-autophagy axis as a fundamental regulatory layer in cancer biology. Nevertheless, the precise molecular mechanisms that connect lactylation and autophagy within the HCC TME, particularly whether they are orchestrated by a specific secreted factor from stromal cells like LSECs, are entirely unknown.

To bridge this critical gap, we employed an integrated multi-omics approach, hypothesizing that key secreted molecules linking lactylation and autophagy could serve as novel prognostic biomarkers and therapeutic targets for HCC. We constructed a lactylation- and autophagy-associated gene signature from public cohorts (TCGA, ICGC) to stratify HCC patients into distinct molecular subtypes with prognostic significance. Through this analysis, C-type lectin domain family 3 member B (CLEC3B, tetranectin), which encodes a secreted protein and belongs to the Ca² ⁺ -binding C-type lectin family [21], was identified as a core component of our prognostic model. Notably, CLEC3B has been reported to be downregulated in several cancers and is associated with poor survival in HCC [22,23]. It also demonstrates a negative correlation with immune cell infiltration and various immune biomarkers [22]. Furthermore, CLEC3B is downregulated in HCC exosomes and has been shown to suppress tumor metastasis and angiogenesis through the AMPK and VEGF signaling pathways [24]. Despite these suggestive findings, the expression pattern, functional significance, and underlying mechanisms of CLEC3B in HCC—particularly from the perspective of LSEC-tumor cell crosstalk—remain largely uncharacterized.

Therefore, to address these questions, this study was designed to validate the specific expression and secretion of CLEC3B from LSECs by leveraging single-cell RNA sequencing data and experimental approaches. We further sought to investigate its clinical relevance and tumor-suppressive functions in HCC. Finally, we mechanistically explored whether CLEC3B exerts its effects through modulation of the autophagy pathway and lactylation levels in cancer cells. Our findings identify CLEC3B as a novel, LSEC-derived tumor suppressor that impedes HCC progression by attenuating autophagy and lactylation, thereby positioning it as a promising prognostic biomarker and a potential therapeutic target.

## 2. Materials and methods

### 2.1. Ethics statement

This study was conducted in accordance with the Declaration of Helsinki and was approved by the Ethics Committee of Tianjin Medical University General Hospital (IRB2024-KY-073). Written informed consent was obtained from all participants.

### 2.2. Data collection and processing

Transcriptomic RNA-sequencing data and corresponding clinical information for hepatocellular carcinoma (HCC) were obtained from The Cancer Genome Atlas (TCGA) LIHC project. We retrieved transcripts per million (TPM)-normalized expression values for both tumor tissues and adjacent normal samples. Only tumor samples with complete overall survival (OS) and clinical annotations were included. Gene expression values were log2(TPM + 1) transformed, and genes detected in fewer than 25% of samples were excluded. For independent validation, transcriptomic and clinical data from an additional HCC cohort were acquired from the International Cancer Genome Consortium (ICGC) under the project ICGC-LIRI-JP. Single-cell RNA-seq datasets were downloaded from the Gene Expression Omnibus (GEO), including data from normal mouse liver (GSE218299), human normal liver (GSE174748) and human HCC tissue (GSE202642). Lactylation-related genes were curated from published studies [25–27]. Autophagy-related genes were retrieved from the Human Autophagy Database (HADb, http://www.autophagy.lu/) and the Autophagy & Disease Database (ATD, https://auto2disease.nwsuaflmz.com/).

### 2.3. Human liver tissue and serum sample acquisition

Paired hepatocellular carcinoma (HCC) tumor tissues, adjacent non-tumorous liver tissues (located >2 cm from the tumor margin), and preoperative peripheral blood samples were prospectively collected from patients who will underwent curative resection between January 1, 2025, and December 31, 2025 at the Department of General Surgery, Tianjin Medical University General Hospital. The diagnosis of HCC was confirmed by postoperative histopathological examination. None of the patients had received any preoperative anticancer therapies (e.g., chemotherapy, radiotherapy, or targeted therapy). A total of 42 patients were included. For comparative analysis of circulating CLEC3B levels in a non-malignant liver disease context, preoperative peripheral blood samples were also collected from an additional 20 patients who underwent surgical resection for liver trauma at the same institution. The diagnosis and indication for surgery were confirmed by imaging and clinical assessment. All tissue samples were immediately snap-frozen in liquid nitrogen after resection and stored at -80°C until RNA or protein extraction. Blood samples were collected in serum separator tubes, allowed to clot at room temperature for 30 minutes, centrifuged at 3000 rpm for 10 minutes, and the resulting serum aliquots were stored at -80°C.

### 2.4. Bioinformatics analysis

#### 2.4.1. Differential gene expression analysis.

Differentially expressed genes (DEGs) between tumor and normal adjacent tissues in the TCGA-LIHC dataset were identified using the DESeq2 package (version 1.36.0) in R [28]. Genes with an adjusted P-value (adj. p) < 0.05 and absolute log2 fold change (|log2FC|) > 1 were considered statistically significant DEGs.

#### 2.4.2. Single-cell RNA-sequencing analysis.

ScRNA-seq data were processed and analyzed using Seurat (v5.2.1) in R [29]. Low-quality cells were excluded based on the following criteria: unique molecular identifiers (UMIs) < 200, mitochondrial gene content > 10%, or outlier expression profiles (>5 median absolute deviations). Gene expression was log-normalized, and the top 3,000 highly variable genes were identified for principal component analysis (PCA). Batch effects were corrected using Harmony. Cell clusters were identified at a resolution of 0.5 via the FindClusters function, and cluster-specific marker genes were detected using FindAllMarkers.

#### 2.4.3. Consensus clustering.

Based on the expression profiles of lactylation- and autophagy-associated DEGs, patients in the TCGA-LIHC cohort were classified using the ConsensusClusterPlus R package (version 1.66.0) [30]. The optimal number of clusters (k) was determined to ensure clustering stability.

#### 2.4.4. Enrichment analysis between subtypes.

Survival analysis between different molecular subtypes was performed using the survival R package (version 3.6-4). Kaplan-Meier (KM) curves were plotted to assess the association between subtypes and patient prognosis. The two subtypes with the most significant survival difference were selected for subsequent enrichment analysis.

DEGs between these two subtypes were identified using DESeq2. Genes were ranked based on log2FC values. Gene Set Enrichment Analysis (GSEA) was conducted using the clusterProfiler package (version 4.10.0) [31]. Kyoto Encyclopedia of Genes and Genomes (KEGG) pathway and Gene Ontology (GO) term enrichment analyses were performed using the MSigDB database (subsets c2.cp.kegg_legacy.v2023.2.Hs.symbols.gmt and c5.go.v2023.2.Hs.symbols.gmt, respectively). Terms with an adjusted P-value < 0.05 were considered significantly enriched.

#### 2.4.5. Immune infiltration analysis.

The relative abundance of 22 immune cell types in each sample was estimated using the CIBERSORT algorithm implemented in the IOBR R package (version 0.99.9) [32]. The Immunophenoscore (IPS) for each TCGA-LIHC sample was predicted, and differences in IPS between subtypes were compared using the Wilcoxon rank-sum test.

The Tumor Immune Dysfunction and Exclusion (TIDE) score (http://tide.dfci.harvard.edu/) was used to predict potential immune checkpoint inhibitor (ICI) benefits. TIDE scores were compared between subtypes using the Wilcoxon test.

The anti-cancer immune status of each sample and the proportions of tumor-infiltrating immune cells across the seven-step cancer-immunity cycle were analyzed and visualized using data from the Tracking Tumor Immunophenotype (TIP, http://biocc.hrbmu.edu.cn/TIP/) portal.

#### 2.4.6. Mutation analysis.

Somatic mutation data for TCGA-LIHC patients were downloaded in Mutation Annotation Format (MAF). The maftools R package (version 2.17.0) [33] was used to calculate the mutation frequency of the top 20 genes and generate a waterfall plot. Tumor Mutation Burden (TMB) was calculated for each tumor sample, and differences in TMB between subtypes were compared using the Wilcoxon rank-sum test and visualized via boxplots.

#### 2.4.7. Construction and evaluation of the risk model.

To elucidate the molecular mechanisms linking lactylation-autophagy crosstalk to patient survival, we compare1d the subtype with the worst prognosis (Cluster 1) and the subtype with the best prognosis (Cluster 2). DEGs between these two subtypes were identified using DESeq2 (adj. p < 0.05 and |log2FC| > 1) and subjected to functional enrichment analysis using clusterProfiler.

To identify prognostic genes for model construction, univariate Cox regression analysis (p < 0.01) was performed on the DEGs to select candidate genes. These candidate genes were further refined using Least Absolute Shrinkage and Selection Operator (LASSO) Cox regression analysis with the glmnet R package (version 4.1-6) [34] in the TCGA-LIHC training set.

A risk score (RiskScore) for each patient in the training set was calculated using the formula: RiskScore = Coef1 * X1 + Coef2 * X2 + … + Coefn * Xn, where Coef represents the coefficient derived from the LASSO Cox regression and X represents the gene expression level. Patients were stratified into high-risk and low-risk groups based on the median RiskScore. The prognostic performance of the model was evaluated using Kaplan-Meier survival analysis and time-dependent Receiver Operating Characteristic (ROC) curves for 1-, 2-, and 3-year overall survival.

#### 2.4.8. Validation of the risk model.

The predictive accuracy of the prognostic model was assessed in the independent ICGC-LIRI-JP validation cohort. Each patient's RiskScore was calculated using the same formula and coefficients from the training set. Patients were classified into high- or low-risk groups based on the median RiskScore from the training set. Kaplan-Meier analysis and time-dependent ROC curves were used to evaluate model performance in the validation set.

#### 2.4.9. Independent prognostic analysis.

To determine whether the RiskScore was an independent prognostic factor in HCC, univariate and multivariate Cox regression analyses were performed on the TCGA-LIHC cohort, incorporating the RiskScore and other clinical features (e.g., age, gender, TNM stage). Factors with p < 0.001 in the multivariate analysis were considered independent prognostic factors. A nomogram was constructed using these independent factors to predict 1-, 2-, and 3-year overall survival probabilities. Calibration curves were plotted to assess the accuracy of the nomogram.

### 2.5. Primary cell isolation

All animal experiments were approved by the laboratory animal welfare and ethics committee of tianjin medical university general hospital. All procedures were performed in accordance with relevant guidelines and regulations. This study is reported in accordance with ARRIVE guidelines (https://arriveguidelines.org). Eight- to ten-week-old male C57BL/6 mice were purchased from Beijing Huafukang Biotech Co., Ltd. Mice were anesthetized by intraperitoneal injection of pentobarbital sodium (50 mg/kg body weight). Primary mouse liver sinusoidal endothelial cells (LSECs) were isolated as previously described [35] with modifications. Briefly, mice were anesthetized, and the liver was perfused sequentially through the portal vein with Hank's buffer without Ca^2+^, followed by Hank's buffer with Ca^2+^ and Mg^2+^ containing 0.8 mg/ml collagenase IV (BS165-1g, Biosharp, China). The liver was then transferred into a 50 ml tube containing 0.8 mg/mL collagenase IV and 10 μg/ml DNase I (BS137–10mg, Biosharp, China). A single-cell suspension was generated by passing the digested tissue through a 100 μm cell strainer and adjusting the volume to 50 ml with DMEM. The suspension was centrifuged at 50 × g for 5 min to pellet hepatocytes. The supernatant containing non-parenchymal cells (NPCs) was centrifuged at 400 × g for 7 min. The NPC pellet was resuspended in 4 ml of 17.6% iodixanol (Y267499, Beyotime, China), gently overlaid with 4 ml of 11.5% iodixanol and 2 ml of DMEM, and centrifuged at 1400 × g for 20 min in a swing-out rotor without brake. The white layer at the interface between the 11.5% and 17.6% iodixanol solutions, containing LSECs, was collected and transferred to a new tube. Cells were incubated with anti-CD146 magnetic beads (130-092-007, Miltenyi Biotec, Germany) for 15 min at 4°C in the dark, washed with PBS, and resuspended in 1 ml PBS. The cell suspension was applied to an MS Column, washed twice with PBS, and LSECs were eluted with 2 ml PBS. The flow-through containing non-LSEC NPCs was also collected. At the end of the perfusion procedure, mice were euthanized by cervical dislocation under deep anesthesia.

### 2.6. Cell lines and culture

All cell lines were cultured under standard sterile conditions in a humidified incubator maintained at 37°C with 5% CO₂. The human hepatocellular carcinoma cell lines Huh7 were purchased from Wuhan Servicebio Technology Co., Ltd. (STCC10102P) and cultured in high-glucose Dulbecco's Modified Eagle Medium (DMEM, Servicebio, GZ10102) supplemented with 10% fetal bovine serum (FBS). The human liver sinusoidal endothelial cell (LSEC) line was obtained from Beijing Bio-BW Biotechnology Co., Ltd. (Catalog No. Bio-129468). These cells were maintained in Endothelial Cell Medium (Servicebio, GZ4567) specifically formulated for endothelial cell growth. Primary mouse LSECs were isolated from C57BL/6 mice as described in detail in Section 2.4 (Primary Cell Isolation). After isolation, the primary mouse LSECs were cultured in the same Endothelial Cell Medium (Servicebio, GZ4567) used for the human LSEC line.

To generate CLEC3B-overexpressing human LSECs, the full-length coding sequence (CDS) of human CLEC3B (NCBI Reference Sequence: NM_003278.3) was synthesized and cloned into the lentiviral expression vector pCDH-CMV-MCS-EF1-CopGFP-T2A-Puro by Synbio Biotechnology Co., Ltd. (Suzou, China). The resulting plasmid, designated pCDH-CMV-MCS-EF1-CopGFP-T2A--Puro-CLEC3B, allows for doxycycline-inducible or constitutive expression of CLEC3B and carries a puromycin resistance gene for selection. For lentivirus production, HEK293T cells were co-transfected with the pCDH-CMV-MCS-EF1-CopGFP-T2A--Puro-CLEC3B plasmid and the packaging plasmids (psPAX2 and pMD2.G) using polyethylenimine (PEI). The viral supernatant was collected at 48 and 72 hours post-transfection, concentrated by ultracentrifugation, and titrated. For transduction, LSEC cells were seeded in 6-well plates and incubated with the concentrated lentivirus at a multiplicity of infection (MOI) of 20 in the presence of 8 μg/mL Polybrene. After 24 hours, the viral-containing medium was replaced with fresh growth medium. To establish stable polyclonal populations, cells were selected with 8 μg/mL puromycin (ST551, Beyotime, China) for at least one week. Successful overexpression of CLEC3B was confirmed by Western blot analysis of whole-cell lysates and ELISA of conditioned media, as shown in Fig 6E and 6F.

### 2.7. Immunofluorescence (IF)

Liver tissues from C57BL/6 mice were fixed with 4% paraformaldehyde for 20 minutes at room temperature, then embedded in optimal cutting temperature (OCT) compound and sectioned at 8 μm thickness using a cryostat (Leica CM1950). Sections were permeabilized with 0.3% Triton X-100 for 10 min and blocked with 3% bovine serum albumin (BSA) for 30 min at room temperature. Primary antibodies against Clec3b (1:200, A4387, Abclonal, China) and CD105 (1:700, GB113377, Servicebio, China) were applied overnight at 4°C. After washing with PBS, sections were incubated with secondary antibodies (1: 300, GB21303, Servicebio, China) for 1 h at room temperature. Nuclei were counterstained with DAPI (G1012, Servicebio, China). Images were acquired using a microscope.

### 2.8. Quantitative real-time PCR

Total RNA was isolated from primary LSECs using TRIZOL reagent, and reverse-transcribed into cDNA with the Prime Script RT reagent kit (R223-01, Vazyme Biotechnology, China). Quantitative PCR (qPCR) assays were performed using PerfectStart Green qPCR SuperMix (AQ601, TransGen Biotech, China). Gene-specific primers were used, with *Actin* as a reference control (S4 Table). The relative mRNA levels of target genes were calculated using the 2-ΔΔCTmethod. At least three samples per genotype were analyzed and the mean relative expression of each gene between groups was used for statistical analysis.

### 2.9. Western blot

Cells were lysed in Radio Immunoprecipitation Assay (RIPA) buffer (G2002, Servicebio, China) containing protease inhibitors (PMSF, G2008, Servicebio, China). Protein concentration was determined using a BCA assay kit (P0010, Beyotime, China). Equal amounts of protein (20–30 μg) were separated by 10% SDS-PAGE and transferred to PVDF membranes. Membranes were blocked with 5% non-fat milk for 1 h and incubated overnight at 4°C with primary antibodies against Clec3b (1:1000, GB113243, Servicebio, China), LC3 (1:1000, GB11124, Servicebio, China), LDHA (1:1000, GB11342, Servicebio, China), P62 (1:1000, GB11239, Servicebio, China), Pan lactylation (1:1000, A23004, Abconal, China) and Actin (1:1500, GB113225, Servicebio, China). After washing with TBST, membranes were incubated with HRP-conjugated secondary antibodies (1:5000, GB23303, Servicebio, China) for 1 h at room temperature. Protein bands were visualized using an ECL detection system (G2161, Servicebio, China) and quantified with ImageJ software.

### 2.10. Cell proliferation assay

Cell proliferation was assessed using the Cell Counting Kit-8 (CCK-8, C0038, Beyotime Biotechnology, China) according to the manufacturer's instructions. Briefly, Huh7 cells were seeded into 96-well plates at a density of 2 × 10^3^ cells per well and allowed to adhere overnight. Cells were then treated with the respective conditioned media or compounds as indicated in the figure legends. For rescue and modulation experiments, the following agents were used: sodium lactate (71718, Sigma-Aldrich, USA) at a final concentration of 30 mM, rapamycin (S1842, Beyotime Biotechnology, China) at 100 nM, and trehalose (Tre, T0167, Sigma-Aldrich, USA) at 50mM. Following 72 hours of treatment, 10 μL of CCK-8 reagent was added to each well, and the plates were incubated at 37°C for 2 hours. The absorbance at 450 nm was measured using a microplate reader. All experiments were performed with at least five replicate wells per condition and repeated independently three times.

### 2.11. Enzyme-linked immunosorbent assay (ELISA)

The concentrations of human CLEC3B protein in serum samples and conditioned media were determined using a commercially available sandwich ELISA kit (Human Tetranectin/CLEC3B ELISA Kit, Catalog No: SYP-H1609, UpingBio, China) according to the manufacturer’s instructions. Briefly, all reagents, samples, and standards were brought to room temperature prior to use. The pre-coated 96-well plate was used directly.

For serum samples: The preoperative serum samples from HCC patients (n = 42) and hepatic hemangioma patients (n = 20), which were collected and processed as described in section 2.2, were diluted 2-fold with the provided sample diluent before assay.

For conditioned media: The culture supernatants were collected from control and CLEC3B-overexpressing human LSECs. The media were centrifuged at 1000 × g for 10 minutes to remove any cellular debris and used undiluted or with appropriate dilution within the kit's detection range.

Assay procedure: First, 100 μL of standard, blank (sample diluent), or prepared sample was added to each appropriate well. The plate was covered and incubated for 90 minutes at 37°C. After incubation, the liquid was discarded, and each well was washed four times with 350 μL of wash buffer. Immediately after the last wash, 100 μL of Biotinylated Detection Antibody working solution was added to each well, followed by a 60-minute incubation at 37°C. The wash step was repeated four times. Subsequently, 100 μL of Horseradish Peroxidase (HRP) Conjugate working solution was added to each well, and the plate was incubated for 30 minutes at 37°C in the dark. After another five wash cycles, 90 μL of Substrate Reagent was added to each well and incubated for 15 minutes at 37°C in the dark. Finally, 50 μL of Stop Solution was added to each well, and the optical density (OD) was measured immediately at 450 nm using a microplate reader.

### 2.12. Statistical analysis

For the bioinformatics analyses presented in this study (including differential gene expression, consensus clustering, survival analysis, GSEA, and immune infiltration estimation), all computational procedures and corresponding figure generation were performed using R software (version 4.3.3) with relevant packages as specified in the respective method sections.

For all *in vitro* experimental data (including qPCR, Western blot quantification and CCK-8 assays), statistical analyses were performed and graphs were generated using GraphPad Prism software (version 9.5, GraphPad Software, USA). Quantitative data are presented as the mean ± standard deviation (SD) from at least three independent experiments. For comparisons between two groups, Student’s t-test (unpaired, two-tailed) was used. For multiple group comparisons, one-way analysis of variance (ANOVA) followed by Tukey’s post hoc test was applied. For all bioinformatics analyses involving multiple hypothesis testing, the Benjamini-Hochberg procedure was used to control the false discovery rate, with adjusted P-values reported where applicable (e.g., DESeq2 for differential expression, clusterProfiler for enrichment analyses). A P-value of less than 0.05 was considered statistically significant (*p < 0.05, **p < 0.01, ***p < 0.001).

## 3. Results

### 3.1. Identification of lactylation- and autophagy-associated molecular subtypes with distinct prognoses

**Fig 1:**
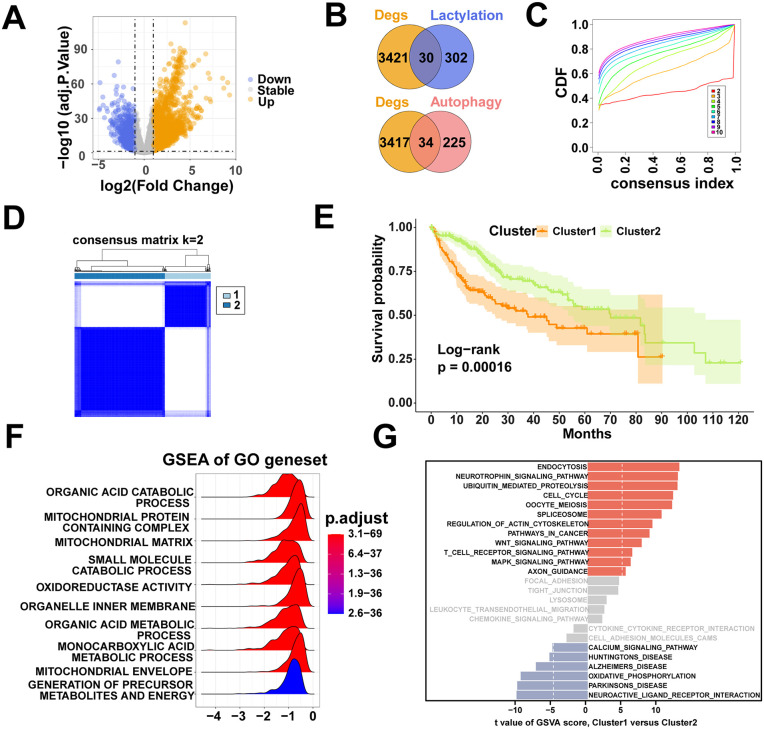
Identification of Lactylation- and Autophagy-Associated DEGs and Patient Stratification in HCC. **(A)** Volcano plot of differentially expressed genes (DEGs) between hepatocellular carcinoma (HCC) tumors and adjacent normal tissues from the TCGA-LIHC cohort. Genes with |log2(Fold Change)| > 1 and adjusted P-value <0.05 are highlighted in yellow (upregulated) and blue (downregulated). **(B)** Venn diagrams showing the intersection of DEGs from (A) with lactylation-associated genes (30 genes) and autophagy-associated genes (34 genes). **(C)** Cumulative distribution function (CDF) curve for consensus clustering of the 64 intersecting genes from **(B)**. The optimal cluster number k = 2 was selected based on minimal change in area under CDF curve. **(D)** Consensus clustering matrix heatmap showing the two molecular subtypes (Cluster 1 and Cluster 2) identified from TCGA-LIHC patients. **(E)** Kaplan-Meier survival curves comparing overall survival between Cluster 1 and Cluster 2 patients. **(F)** Gene Set Enrichment Analysis (GSEA) of Gene Ontology (GO) biological processes for genes differentially expressed between Cluster 1 and Cluster 2. **(G)** GSVA enrichment pathways in Cluster1 and Cluster2 subgroups. Differential expression analysis was performed using DESeq2. Survival difference was assessed by log-rank test. GSEA and GSVA were conducted using clusterProfiler and GSVA packages in R, respectively.

To identify molecular signatures associated with lactylation and autophagy in hepatocellular carcinoma (HCC), we analyzed transcriptomic data from the TCGA-LIHC cohort. Differential gene expression analysis comparing tumor tissues (n = 371) to adjacent normal tissues (n = 50) revealed 3541 significantly differentially expressed genes (DEGs) meeting the thresholds of |log₂ fold change| > 1 and adjusted P-value <0.05, visualized in a volcano plot (Fig 1A).

Subsequently, these DEGs were intersected with curated gene sets. Intersection with a set of experimentally validated lactylation-Associated genes (S1 Table) yielded 30 overlapping genes. Similarly, intersection with a canonical set of autophagy-associated genes (S1 Table) yielded 34 overlapping genes (Fig 1B, S2 Table). As described in the methods, we performed unsupervised consensus clustering analysis on the TCGA-LIHC cohort based on the expression profiles of these 64 genes. The clustering results were compared, and the cumulative distribution function (CDF) plot displayed the consensus distribution for each K (Fig 1C). According to the evaluation of within-group average consistency, K = 2 ranked highest (S1C Fig). Based on this, TCGA-LIHC patients were divided into two distinct molecular subgroups: Cluster1 and Cluster2 (Fig 1D, S3 Table). Prognostic significance of these molecular clusters was assessed using Kaplan-Meier survival analysis. Strikingly, patients assigned to Cluster 2 exhibited significantly longer overall survival compared to those in Cluster 1 (log-rank p = 0.00016) (Fig 1E). To elucidate the biological pathways underlying the prognostic difference between clusters, we performed differential expression analysis between Cluster 1 and Cluster 2. S1B Fig illustrates a heatmap of the top 25 most significantly upregulated and downregulated differentially expressed genes. Gene Set Enrichment Analysis (GSEA) of Gene Ontology (GO) Biological Processes using these cluster-defining DEGs revealed significant enrichment of terms related to “ENDOCYTOSIS”, “NEUROTROPHIN SIGNALING PATHWAY”, NEUROACTIVE LIGAND RECEPTOR INTERACTION” and “PARKINSONS DISEASE” in Cluster 1 compared to Cluster 2 (Fig 1F). S1D Fig demonstrates that the KEGG enrichment analysis identified enriched pathways, including oxidative phosphorylation, Parkinson's disease, retinol metabolism, and drug metabolism - cytochrome P450. Furthermore, Gene Set Variation Analysis (GSVA) was performed on the training dataset using a threshold of FDR < 0.05. The pathway outcomes comparing Cluster1 samples are presented in Fig 1G, revealing enrichment in pathways such as “Endocytosis,” “Neurotrophin Signaling Pathway,” “Neuroactive Ligand-Receptor Interaction,” and “Parkinson's Disease.” Subsequent correlation analysis of molecular pathways demonstrated substantial positive correlations between “Oxidative Phosphorylation” and “Parkinson's Disease,” as well as between “Cell Adhesion Molecules (CAMs)” and “Chemokine Signaling Pathway” (S1E Fig). Together, these findings establish two molecular subtypes of HCC defined by lactylation- and autophagy-associated gene expression, which are strongly correlated with divergent patient outcomes and enriched in distinct biological pathways.

### 3.2. Immune infiltration profiles and somatic mutation characteristics of HCC molecular subgroups

**Fig 2:**
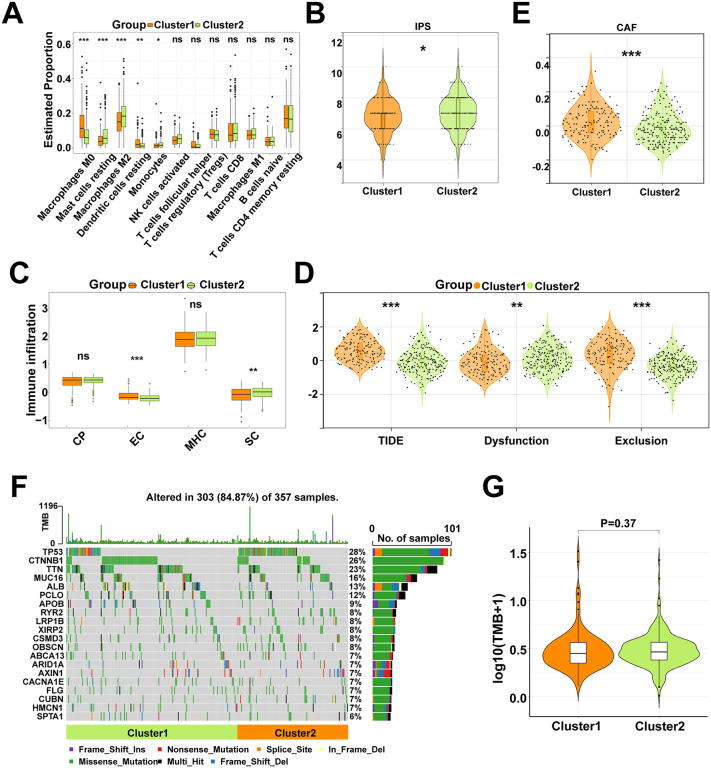
Immune Infiltration Profiles and Somatic Mutation Characteristics of HCC Molecular Subgroups. **(A)** Box plots showing the relative abundance of 12 immune cell types with significant infiltration differences between Cluster 1 and Cluster 2 (CIBERSORT analysis). **(B)** Immunophenoscore (IPS) comparison between the two subtypes. **(C)** Comparison of cytolytic activity (CP), effector cells (EC), MHC molecules (MHC), and immune checkpoints (SC) scores between subtypes. **(D)** Tumor Immune Dysfunction and Exclusion (TIDE) scores, Dysfunction scores, and Exclusion scores between subtypes. **(E)** Differences in CAF scores between different subtypes. **(F)** Waterfall plot showing the mutation landscape of top 20 frequently mutated genes in both subtypes. **(G)** Tumor mutational burden (TMB) comparison between subtypes. * p < 0.05, ** p < 0.01, *** p < 0.001.

Evaluation of 22 immune cell types using CIBERSORT identified 12 cell types with non-negligible abundance (Fig 2A). Among these, the infiltration levels of Dendritic cells resting, Macrophages M0, Macrophages M2, and Mast cells resting were significantly different between Cluster 1 and Cluster 2 (p < 0.05). Further immunophenotypic characterization demonstrated subtype-specific immunogenicity and potential therapeutic vulnerabilities. Analysis using the Immunophenoscore (IPS) revealed significantly different scores between the clusters (Fig 2B). Evaluation of critical immunomodulatory components showed that Cytolytic Potential (CP) and MHC molecule expression (MHC) scores were comparable between subgroups, while Effector Cells (EC), Immune Checkpoints (SC), Dysfunction, and Exclusion scores, as well as the overall Tumor Immune Dysfunction and Exclusion (TIDE) score, all exhibited significant differences (Fig 2C, D). Furthermore, analysis of Cancer-Associated Fibroblast (CAF) infiltration (Fig 2E) demonstrated significantly lower CAF levels in Cluster 2 compared to Cluster 1. Visualization of the individual sample-level anti-cancer immune status across subtypes highlighted the heterogeneity within clusters (S2A Fig). Assessment of the seven-step cancer-immunity cycle revealed distinct proportions of tumor-infiltrating immune cells participating in each step between Cluster 1 and Cluster 2 (S2B Fig). Furthermore, expression analysis of established immune checkpoint genes showed significantly different levels for all genes examined except HLA-B (S2C Fig). Analysis of the top 20 most frequently mutated genes demonstrated distinct mutation patterns between the subtypes (Fig 2F). However, calculation of Tumor Mutational Burden (TMB) showed no significant difference in overall mutation load between Cluster 1 and Cluster 2 (Fig 2G). These results reveal significant differences in immune cell infiltration, immunophenotypic scores, and immune checkpoint expression between the two subtypes, while indicating no substantial difference in tumor mutational burden.

### 3.3. Construction and validation of a prognostic 5-gene signature based on HCC molecular subtypes

**Fig 3:**
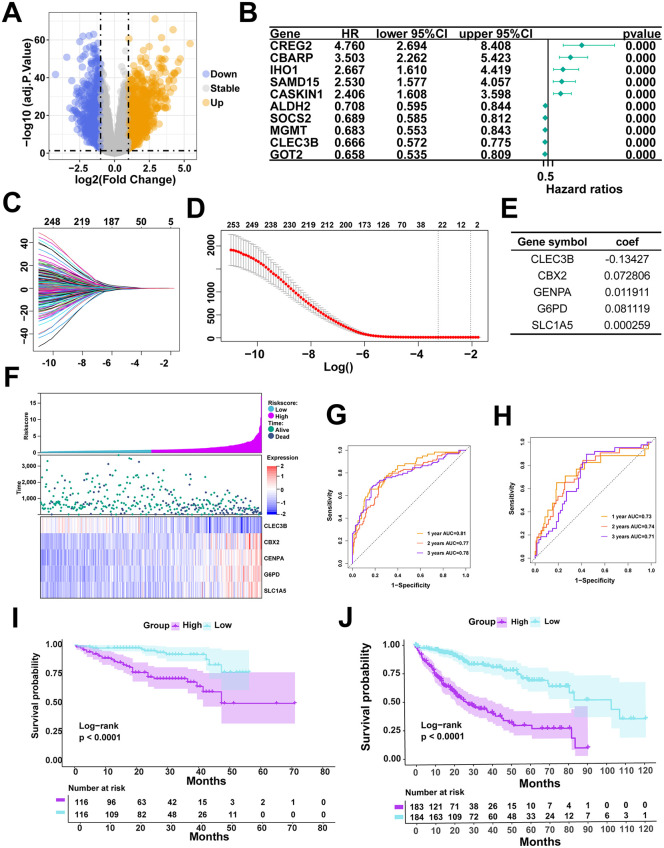
Construction and Validation of a Prognostic 5-Gene Signature Based on HCC Molecular Subtypes. **(A)** Volcano plot of differentially expressed genes between Cluster 1 and Cluster 2 (2001 DEGs with 1264 upregulated and 737 downregulated in Cluster 1 vs Cluster 2). **(B)** Forest plot showing the top 5 and bottom 5 prognostic genes from univariate Cox regression analysis of the 2001 DEGs. **(C)** LASSO coefficient profiles of the 254 candidate prognostic genes. **(D)** Cross-validation for tuning parameter selection in the LASSO Cox regression model. **(E)** Final five genes selected by LASSO Cox regression with their coefficients. **(F)** Risk score distribution, patient survival status, and expression heatmap of the five prognostic genes in the TCGA-LIHC cohort. **(G-H)** Time-dependent ROC curves analysis showing the predictive accuracy of the risk score for 1-, 2-, and 3-year overall survival in **(G)** TCGA-LIHC and **(H)** ICGC-LIRI-JP cohorts. **(I-J)** Kaplan-Meier survival analysis for high- and low-risk groups in **(I)** TCGA-LIHC and **(J)** ICGC-LIRI-JP cohorts.

To elucidate the transcriptional drivers underlying the prognostic differences between the poor-prognosis subtype (Cluster 1) and favorable-prognosis subtype (Cluster 2) identified in Fig 1, we performed differential gene expression analysis. DESeq2 analysis identified 2001 significantly differentially expressed genes (DEGs) between Cluster 1 and Cluster 2 (1264 upregulated, 737 downregulated in Cluster 1 vs. Cluster 2; thresholds: |log₂FC| > 1, adj. P-value < 0.05), visualized in a volcano plot (Fig 3A). Hierarchical clustering based on these DEGs confirmed distinct transcriptional profiles between the subtypes (S3A Fig). Gene Ontology (GO) enrichment analysis (S3B Fig) identified significant terms including “alcohol metabolic process,” “lipid catabolic process,” “secretory granule lumen,” “cytoplasmic vesicle lumen,” “amide binding,” and “metal ion transmembrane transporter activity.” Kyoto Encyclopedia of Genes and Genomes (KEGG) pathway analysis (S3C Fig) showed significant enrichment for pathways such as “Chemical carcinogenesis - DNA adducts,” “PPAR signaling pathway,” “Complement and coagulation cascades,” and “Tryptophan metabolism.” To identify genes with direct prognostic relevance, the 2001 DEGs were subjected to univariate Cox proportional hazards regression analysis for overall survival. This analysis yielded 254 candidate prognostic genes significantly associated with survival (P-value < 0.05). The top 5 genes with the highest Hazard Ratios (HRs; poor prognosis) and the bottom 5 genes with the lowest HRs (favorable prognosis) were visualized in a forest plot (Fig 3B). To refine the prognostic signature and avoid overfitting, we employed machine learning. Using the Least Absolute Shrinkage and Selection Operator (LASSO) Cox regression on the 254 candidate genes within the TCGA-LIHC cohort, we optimized the penalty parameter (lambda) via 10-fold cross-validation (Fig 3C, D). This resulted in the selection of a parsimonious 5-gene signature: CLEC3B, CBX2, CENPA, G6PD, and SLC1A5 (Fig 3E). Kaplan-Meier analysis confirmed that high expression of CBX2, CENPA, G6PD, and SLC1A5 was significantly associated with poor prognosis (log-rank p < 0.05), while high expression of CLEC3B was associated with favorable prognosis (S3D-H Fig). A prognostic risk score (Riskscore) was calculated for each patient based on the expression levels of the 5 genes weighted by their LASSO regression coefficients. Visualization of Riskscore, survival status, and gene expression patterns revealed a clear association between higher Riskscore and poorer survival outcomes (Fig 3F). The prognostic performance of the Riskscore was rigorously evaluated. Time-dependent Receiver Operating Characteristic (ROC) curve analysis demonstrated strong predictive accuracy for 1-, 2-, and 3-year overall survival in both the TCGA-LIHC cohort (AUCs = 0.81, 0.77, 0.78; Fig 3G) and the independent Validation cohort (ICGC-LIRI-JP, AUCs = 0.73,0.74,0.71; Fig 3H), with all AUCs > 0.7. Patients were stratified into High-Risk and Low-Risk groups based on the median Riskscore. Kaplan-Meier survival analysis confirmed that patients in the High-Risk group had significantly poorer overall survival compared to the Low-Risk group in both the TCGA-LIHC cohort (Fig 3I) and the ICGC-LIRI-JP cohort (Fig 3J). In summary, we successfully developed and validated a robust 5-gene prognostic signature that effectively stratifies HCC patients into high- and low-risk groups with significant differences in overall survival.

### 3.4. Construction of integrated prognostic models for HCC patients

**Fig 4:**
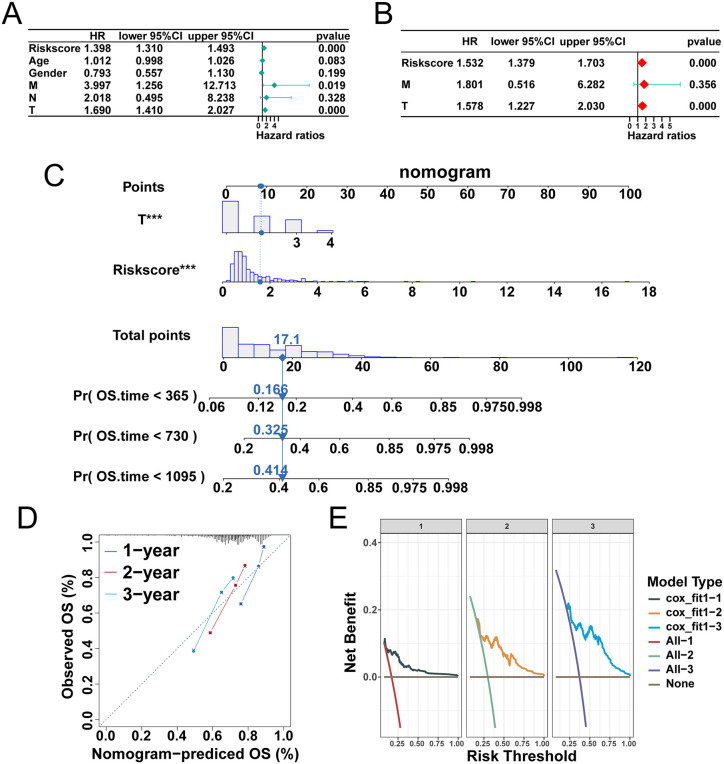
Construction of integrated prognostic models for HCC patients. **(A)** Forest plot of univariate Cox regression analysis for the risk score and clinical characteristics in the TCGA-LIHC cohort. **(B)** Forest plot of multivariate Cox regression analysis identifying independent prognostic factors. **(C)** Nomogram incorporating T stage and risk score for predicting 1-, 2-, and 3-year overall survival probability. **(D)** Calibration curve of the risk model nomogram. **(E)** Decision Curve Analysis.

In the TCGA-LIHC cohort, univariate Cox analysis of the prognostic model risk score (RiskScore) and clinical factors revealed that the clinical features T, M, and RiskScore were all significantly associated with prognosis (P < 0.05) (Fig 4A). The proportional hazards (PH) assumption test indicated that RiskScore was significant, while the clinical features T and M were not. Through stepwise multivariate Cox regression, T and RiskScore were found to be significantly prognostic (Fig 4B). Finally, a clinical risk model nomogram incorporating these two factors, T and RiskScore, was constructed (Fig 4C-E). The integration of the risk score with clinical staging into a nomogram provides a clinically applicable tool for improved prognostic prediction in HCC patients.

### 3.5. CLEC3B is predominantly expressed by liver sinusoidal endothelial cells and is downregulated in HCC

To precisely define the cellular origin and expression dynamics of CLEC3B in the hepatic microenvironment, we performed an integrated analysis of single-cell RNA sequencing (scRNA-seq) data from normal livers.

**Fig 5:**
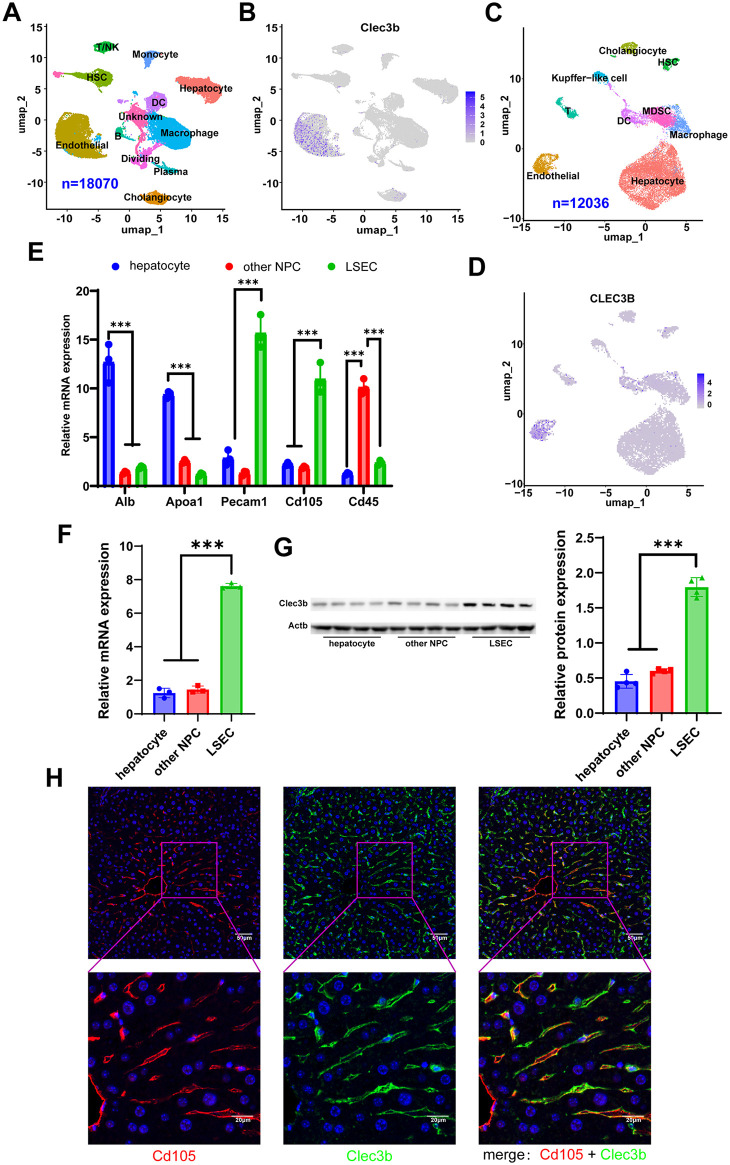
Single-Cell and Experimental Validation of CLEC3B as a Liver Sinusoidal Endothelial Cell-Specific Gen. (A) Uniform Manifold Approximation and Projection (UMAP) visualization of 18070 cells from normal mouse liver tissue (GSE218299) clustered into 12 cell populations. **(B)** FeaturePlot showing Clec3b expression pattern in mouse liver cells. **(C)** UMAP visualization of 12,036 cells from normal human liver tissue (GSE174748) clustered into 9 cell populations. **(D)** FeaturePlot showing CLEC3B expression pattern in human liver cells. **(E)** Quantitative PCR analysis of cell marker gene in primary mouse hepatocytes (HCs), liver sinusoidal endothelial cells (LSECs), and other non-parenchymal cells (NPCs). **(F)** Quantitative PCR analysis of Clec3b expression in HCs, LSECs, and other NPCs. **(G)** Western blot analysis of Clec3b protein expression in HCs, LSECs, and other NPCs. **(H)** Immunofluorescence co-staining of Clec3b (green) and Cd105 (red, LSEC marker) in normal mouse liver tissue. Nuclei were counterstained with DAPI (blue). Data are presented as mean ± SD. *p < 0.05, **p < 0.01, ***p < 0.001.

We first analyzed scRNA-seq data from normal mouse liver (GSE218299, 18,070 high-quality cells), which identified 12 distinct cell populations (Fig 5A). FeaturePlot visualization revealed predominant enrichment of Clec3b expression within endothelial cells (Fig 5B). Consistent with this, analysis of human normal liver scRNA-seq data (GSE174748, 12,036 cells clustered into 9 populations) also demonstrated high expression of CLEC3B specifically in endothelial cells (Fig 5C, D). Given that liver sinusoidal endothelial cells (LSECs) represent the major endothelial subtype in the liver sinusoids and emerged as a key candidate source from our bioinformatic analysis, we proceeded to isolate primary mouse hepatocytes (HCs), LSECs, and other non-parenchymal cells (NPCs) for validation. Prior to assessing CLEC3B expression, we confirmed the purity of these isolated cell populations. qPCR analysis of established cell-type-specific markers showed that hepatocyte markers (Alb, Apoa1) were predominantly and significantly enriched in HCs, endothelial markers (Pecam1, Cd105) were highly expressed in LSECs, and the pan-immune marker Cd45 was primarily detected in the other NPC fraction, which is largely composed of immune cells (Fig 5E). This confirmed the successful and specific isolation of our target cell types. With the purity of our cellular preparations established, we then quantified Clec3b expression. Both mRNA and protein levels of Clec3b were significantly higher in LSECs compared to hepatocytes and other NPCs (Fig 5F, G). Immunofluorescence co-staining of normal mouse liver sections for CD105 (an LSEC marker) and Clec3b further corroborated their co-localization, solidifying LSECs as the primary cellular source of Clec3b *in vivo* (Fig 5H). Collectively, our multi-species, multi-condition scRNA-seq analysis, complemented by experimental validation, unequivocally identifies LSECs as the key cellular source of CLEC3B.

### 3.6. Tumor-suppressive role of LSEC-secreted CLEC3B in HCC via regulation of autophagy and lactylation

**Fig 6:**
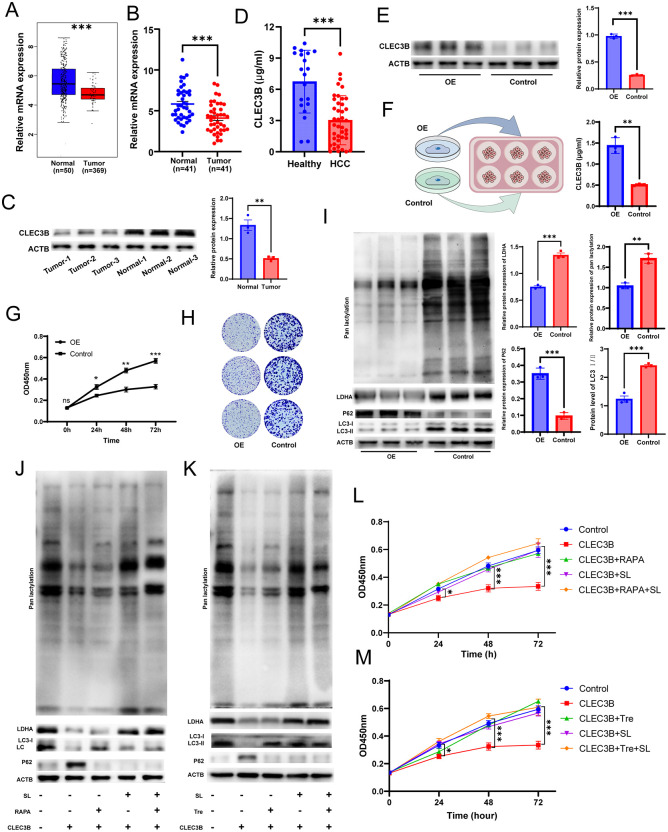
Tumor-Suppressive Role of LSEC-Secreted CLEC3B in HCC via Regulation of Autophagy and Lactylation. **(A)** CLEC3B mRNA expression in TCGA-LIHC normal (n = 50) and HCC (n = 369) tissues. (B) qPCR validation of CLEC3B in an independent cohort of 41 paired HCC/normal tissues. **(C)** Western blot of CLEC3B protein in three representative paired tissues. **(D)** ELISA of serum CLEC3B levels in HCC patients (n = 42) versus liver trauma patients (n = 20, control). **(E)** Western blot confirming CLEC3B overexpression (OE) in human LSECs. **(F)** ELISA of secreted CLEC3B in conditioned media from control and OE-LSECs. **(G)** CCK-8 assay of Huh7 cell proliferation after 72h treatment with Control or OE-LSEC conditioned media. **(H)** Colony formation assay of Huh7 cells. **(I)** Western blot analysis of LDHA, pan lactylation, LC3, and p62 in Huh7 cells treated with conditioned media for 48h. **(J, K)** Western blot analysis of the indicated proteins in Huh7 cells subjected to rescue experiments with conditioned media plus: sodium lactate (SL, 30 mM), rapamycin (RAPA, 100 nM), or trehalose (Tre, 50 mM). **(L, M)** Corresponding CCK-8 proliferation assays for the rescue experiments. Data are presented as mean ± SD. *p < 0.05, **p < 0.01, ***p < 0.001.

Building upon the LSEC-specific expression of CLEC3B established in Fig 5, we investigated its potential tumor-suppressive role and underlying mechanisms. Analysis of the TCGA-LIHC dataset revealed that CLEC3B mRNA expression was significantly lower in HCC tumor tissues (n = 369) compared to adjacent normal liver tissues (n = 50) (Fig 6A). This finding was validated in an independent clinical cohort from Tianjin Medical University General Hospital (n = 41 patient-matched pairs), where CLEC3B mRNA levels, as measured by qPCR, were also significantly downregulated in tumor tissues (Fig 6B). Western blot analysis of three representative patient pairs confirmed the consistent reduction of CLEC3B protein expression in HCC tissues (Fig 6C).

Given the predominant expression of CLEC3B in LSECs and its secreted nature, we hypothesized that LSEC-derived CLEC3B acts as a tumor suppressor. To test this, we measured serum CLEC3B protein levels via ELISA. The results demonstrated that serum CLEC3B concentrations were significantly lower in HCC patients compared to individuals undergoing surgery for liver trauma (a non-malignant liver condition control) **(Fig 6D).** Furthermore**,** we generated a human LSEC line with stable overexpression of CLEC3B (OE) via lentiviral transduction, which was confirmed by Western blot (**Fig 6E**). ELISA of conditioned media from these cells demonstrated significantly higher secretion of CLEC3B from OE-LSECs compared to control-LSECs (**Fig 6F**). We next assessed the functional impact of LSEC-secreted CLEC3B on HCC cell proliferation. Treatment of human Huh7 HCC cells with conditioned media from OE-LSECs significantly suppressed cell proliferation compared to media from control-LSECs, as measured by CCK-8 assay (**Fig 6G**) and colony formation assay (**Fig 6H**).

Given the original context of CLEC3B identification through autophagy- and lactylation-associated gene signatures, we investigated whether these pathways were involved in its tumor-suppressive mechanism. We first examined its impact on lactate metabolism. qPCR analysis confirmed that LDHA mRNA levels were significantly downregulated in Huh7 cells treated with conditioned media from CLEC3B-overexpressing LSECs (**S4A Fig**). Next, Western blot analysis validated this finding at the protein level, showing reduced LDHA expression alongside a decrease in global protein lactylation (Pan lactylation). Concomitantly, analysis of autophagy markers revealed a significant decrease in the LC3-II/LC3-I ratio and an accumulation of p62 ((**Fig 6I**)), both indicating suppressed autophagic flux.

To mechanistically dissect this dual suppression, we utilized pharmacological agents known to activate lactylation or autophagy. First, we determined the optimal concentration of sodium lactate (SL) for inducing protein lactylation in Huh7 cells without causing cytotoxicity, establishing 30 mM as the effective dose for subsequent experiments (S4B Fig). Next, we confirmed the intrinsic activity of the chosen agents. In control Huh7 cells treated with conditioned media from Vector-LSECs (which lack CLEC3B overexpression), addition of the mTOR-dependent autophagy inducer rapamycin (RAPA, 100 nM), the mTOR-independent inducer trehalose (Tre, 50 mM), or SL (30 mM) each effectively enhanced protein lactylation, autophagic flux (increased LC3-II/LC3-I ratio, decreased p62), and cell proliferation compared to the Vector control alone (S4C–S4F Fig). This verified that RAPA, Tre, and SL are potent general activators of their respective target pathways in our experimental system.

Having established the efficacy of these agents, we then tested their ability to counteract the suppressive effects of LSEC-secreted CLEC3B. Huh7 cells were treated with conditioned media from CLEC3B-overexpressing LSECs and divided into five groups: (i) CLEC3B alone, (ii) CLEC3B+RAPA, (iii) CLEC3B+Tre, (iv) CLEC3B+SL, and (v) CLEC3B+RAPA+SL/Tre. Western blot analysis (Fig 6J, K) revealed that, compared to untreated control, the CLEC3B group exhibited the expected suppression of LDHA, global lactylation, and autophagic flux. Both RAPA and Tre restored the LC3-II/LC3-I ratio but did not reverse the suppression of lactylation or LDHA. In striking contrast, SL not only restored lactylation and LDHA levels but also concurrently restored autophagic flux. Corresponding CCK-8 assays (Fig 6L, M) demonstrated that the significant proliferation inhibition caused by CLEC3B-conditioned media was fully rescued in all groups co-treated with RAPA, Tre, or SL.

Collectively, these data establish a clear functional antagonism: CLEC3B suppresses, while RAPA/Tre/SL activate, the lactylation-autophagy axis. More importantly, the unidirectional rescue pattern-where restoring lactylation (via SL) also restored autophagy, but restoring autophagy (via RAPA/Tre) did not affect lactylation-indicates that within the mechanism of CLEC3B, the suppression of lactylation acts upstream of and contributes to the inhibition of autophagy.

## 4. Discussion

Our study delineates a novel tumor-suppressive axis within the hepatocellular carcinoma (HCC) microenvironment, centered on the liver sinusoidal endothelial cell (LSEC)-derived secreted protein, CLEC3B. By integrating multi-omics analyses with functional validation, we demonstrate that CLEC3B acts as a critical stromal-derived factor that concurrently suppresses protein lactylation and autophagic flux in HCC cells, thereby inhibiting tumor progression. These findings reposition the LSEC secretome as a key regulator of HCC metabolism and cell survival, offering fresh insights into stromal-epithelial crosstalk.

The role of protein lactylation, a metabolite-derived post-translational modification, in promoting HCC is becoming increasingly clear. Recent work has elucidated a multi-faceted pro-tumorigenic network: lactylation stabilizes oncoproteins like RET to drive proliferation [36]; it establishes a self-reinforcing positive feedback loop through AKR1B10 and LDHA to enhance glycolysis and confer targeted therapy resistance [37]; and it upregulates major vault protein (MVP) via histone modification, leading to PD-L1 stabilization and immune checkpoint inhibitor resistance [38]. This collective evidence establishes lactylation as a central hub coordinating growth signals, metabolic adaptation, and immune evasion in HCC.

Building on this concept, our work identifies CLEC3B as a pivotal upstream negative regulator of this oncogenic lactylation network. We pinpoint LSECs, rather than parenchymal cells, as the primary source of CLEC3B in the liver and show that its reduction in HCC results from a loss of LSEC abundance. Functionally, CLEC3B secretion from LSECs acts upon HCC cells to significantly reduce global protein lactylation levels. Mechanistically, this is achieved, at least in part, through the suppression of LDHA expression and activity—strikingly, the very enzyme that serves as the common metabolic amplifier in lactylation-driven pathways such as the AKR1B10-LDHA axis [37]. This positions CLEC3B as a unique microenvironmental signal capable of dampening the lactylation network at its metabolic source.

**Fig 7:**
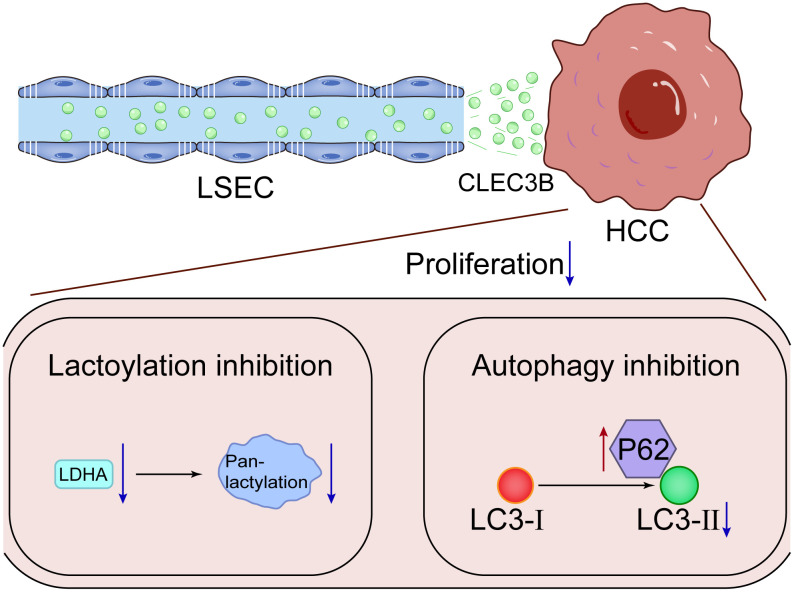
Schematic model illustrating the tumor-suppressive mechanism of LSEC-derived CLEC3B in hepatocellular carcinoma (HCC).

An equally significant finding is the concurrent suppression of autophagic flux by CLEC3B. This dual inhibition prompts a key mechanistic question regarding the relationship between these two processes. Our functional rescue experiments provide pivotal insight: restoring lactylation in CLEC3B-expressing cells rescued both lactylation and autophagic activity, whereas directly activating autophagy failed to restore lactylation. This unidirectional rescue profile strongly indicates that, within this signaling axis, lactylation operates upstream of autophagy and is a critical driver of autophagic flux. We therefore propose that CLEC3B’s primary action of reducing lactate availability diminishes protein lactylation, which in turn leads to attenuated autophagy. The molecular basis for this hierarchy may involve lactylation-dependent modification of core autophagy components. Plausible targets include key transcriptional regulators such as TFEB, where lactylation could enhance its activity to promote lysosomal biogenesis, or kinases within the autophagy initiation machinery like ULK1. This refined model positions CLEC3B as disrupting a coherent lactylation-autophagy hierarchy—a metabolic-sensing circuit where lactylation transduces the loss of stromal-derived CLEC3B signals into diminished autophagic pro-survival responses in HCC cells. Together, these findings delineate a novel LSEC-CLEC3B-lactylation-autophagy axis, which is schematically summarized in Fig 7. It should be noted that the crosstalk between lactylation and autophagy is a nascent research area, and the broader biological foundation remains under active investigation. As such, our proposed model represents an initial framework that will require refinement as the molecular details of this interplay are further elucidated by the field.

The marked downregulation of CLEC3B in the HCC microenvironment prompts the question of its upstream regulation. While our study does not directly address this, we can speculate based on the established pathophysiology of LSEC “capillarization.” This phenotypic transformation, a hallmark of chronic liver disease and HCC, is driven by factors such as hypoxia (via HIF signaling), inflammatory cytokines (e.g., TGF-β, VEGF), and likely paracrine signals from tumor cells themselves [39–42]. These cues may collectively reprogram LSECs, suppressing the expression of homeostatic factors like CLEC3B. Future work to delineate the dominant signal responsible for CLEC3B silencing could reveal strategies to therapeutically restore this tumor-suppressive function.

Our study has several limitations that point to future directions. First, the molecular mechanism by which CLEC3B exerts its tumor-suppressive effects remains incompletely defined. The specific receptor for CLEC3B on the surface of HCC cells has not been identified, and the intracellular signaling cascade linking receptor engagement to LDHA transcriptional suppression is unknown. Second, although our rescue experiments establish a hierarchical relationship in which lactylation acts upstream of autophagy, the direct molecular targets of lactylation within the autophagic machinery—such as TFEB, ULK1, or other core components—have not been experimentally validated. Third, we cannot formally exclude the possibility that CLEC3B modulates other metabolic or signaling pathways beyond lactylation and autophagy, and the rescue experiments in Fig 6I–J are correlative in nature; while they demonstrate that the anti-proliferative effect of CLEC3B depends on these pathways, they do not distinguish between direct molecular targeting and indirect downstream consequences. Broader metabolomic or phosphoproteomic profiling, coupled with targeted lactylomics, would be required to assess both the specificity of CLEC3B's effects and the precise molecular linkages within the lactylation-autophagy axis. Furthermore, the in vivo relevance of our findings warrants validation using LSEC-specific conditional knockout models. Our clinical validation was performed in a relatively small cohort (n = 41) from a single institution, and future multi-center studies with larger sample sizes are needed to confirm the prognostic and diagnostic utility of serum CLEC3B. Translational efforts should explore the therapeutic potential of recombinant CLEC3B protein or strategies to reactivate its expression in the tumor microenvironment. Specifically, experiments such as co-immunoprecipitation coupled with mass spectrometry or genome-wide CRISPR-Cas9-based receptor screening could be employed to identify the CLEC3B receptor. Once identified, detailed dissection of the downstream signaling pathway—from receptor activation to LDHA regulation—would significantly advance our understanding of this tumor-suppressive axis.

Several key questions arising from this study warrant focused investigation. First, the identification of the CLEC3B receptor on HCC cells is of paramount importance. Computational approaches mining co-expression networks or published protein-protein interaction datasets—such as the STRING database or BioPlex—could generate candidate receptor lists for prioritized experimental validation. Second, the use of CLEC3B-overexpressing LSEC-specific transgenic mice or LSEC-conditional Clec3b knockout models would provide critical in vivo evidence for the tumor-suppressive function of LSEC-derived CLEC3B, and would allow assessment of its impact on tumor initiation, growth, and metastasis in physiologically relevant contexts. Third, given the emerging nature of the lactylation-autophagy crosstalk field, systematic profiling of the lactylome in CLEC3B-treated versus untreated HCC cells—using quantitative mass spectrometry—could pinpoint the specific lactylation sites on autophagy-related proteins that mediate the functional connection between these two processes. Finally, exploring whether recombinant CLEC3B protein administration or LSEC-targeted gene therapy to restore CLEC3B expression could suppress HCC growth in preclinical models would represent a logical translational extension of our findings.

In conclusion, we identify CLEC3B as a keystone LSEC-derived factor that constrains HCC progression by dually inhibiting the lactylation and autophagy pathways. This work underscores the critical role of vascular stroma in metabolic regulation and nominates the CLEC3B-LDHA axis as a potential therapeutic target for reinstating metabolic control and impairing tumor cell survival in HCC.

## Supporting information

S1 Fig Identification of Lactylation- and Autophagy-Associated DEGs and Patient Stratification in HCC. (A) Heatmap of 50 differentially expressed genes, where warm colors represent high expression and cool colors represent low expression. (B) Heatmap of differentially expressed genes among different subgroups, with warm colors representing high expression and cool colors representing low expression. (C) Histogram of consensus values for each K from K = 2 to K = 10 (D) Kyoto Encyclopedia of Genes and Genomes (KEGG) pathway enrichment analysis for genes differentially expressed between Cluster 1 and Cluster 2. (E) Relevance map of molecular pathways.
(DOCX)

S2 Fig Immune Infiltration Profiles and Somatic Mutation Characteristics of HCC Molecular Subgroups. (A) Box plots showing the relative abundance of 12 immune cell types with significant infiltration differences between Cluster 1 and Cluster 2 (CIBERSORT analysis). (B) Immunophenoscore (IPS) comparison between the two subtypes. (C) Comparison of cytolytic activity (CP), effector cells (EC), MHC molecules (MHC), and immune checkpoints (SC) scores between subtypes. (D) Tumor Immune Dysfunction and Exclusion (TIDE) scores, Dysfunction scores, and Exclusion scores between subtypes. (E) Differences in CAF scores between different subtypes. (F) Waterfall plot showing the mutation landscape of top 20 frequently mutated genes in both subtypes. (G) Tumor mutational burden (TMB) comparison between subtypes. *p < 0.05, **p < 0.01, ***p < 0.001.
(DOCX)

S3 Fig Construction and Validation of a Prognostic 5-Gene Signature Based on HCC Molecular Subtypes. (A) Heatmap of 50 differentially expressed genes, warm colors represent high expression and cool colors represent low expression. (B) GO enrichment plot of subtype-specific differentially expressed genes. Red, blue, and yellow represent BP, CC, and MF terms, respectively. The x-axis represents the enriched pathways, while the y-axis indicates the number of genes enriched in each pathway. (C) KEGG enrichment analysis dendrogram. The size of the circles represents the number of genes, and the color represents the adjusted P-value. Warmer colors correspond to smaller values, while cooler colors indicate larger values. (D-H) KM curve of high and low groups of characteristic genes.
(DOCX)

S4 Fig Dose effect of sodium lactate on lactylation and specificity validation of CLEC3B-dependent rescue. (A) qPCR analysis of LDHA mRNA levels in Huh7 cells treated with Control or OE-LSEC conditioned media. (B) Dose-dependent induction of protein lactylation by sodium lactate in Huh7 cells. (C, D) Western blot analysis of the indicated proteins in Huh7 cells subjected to rescue experiments with conditioned media plus: sodium lactate (SL, 30 mM), rapamycin (RAPA, 100 nM), or trehalose (Tre, 50 mM). (E, F) Corresponding CCK-8 proliferation assays for the rescue experiments. Data are presented as mean ± SD. *p < 0.05, **p < 0.01, ***p < 0.001.
(DOCX)

S1 Table Lists of lactylation-associated genes and autophagy-associated genes used for analysis. (XLSX)

S2 Table Overlapping differentially expressed genes (DEGs) related to lactylation and autophagy. (XLSX)

S3 Table Consensus clustering assignment of TCGA-LIHC patients into molecular subtypes (Cluster 1 and Cluster 2). (XLSX)

S4 Table Sequences of primers used for quantitative real-time PCR. (XLSX)
