# Supplementary figures and images for: A lactylation- and autophagy-associated prognostic signature reveals LSEC-derived CLEC3B as a novel mediator of hepatocellular carcinoma suppression
Source: PLoS Comput Biol. 2026 Jun 22;22(6):e1014426. doi: 10.1371/journal.pcbi.1014426 (PMC13309010; doi:10.1371/journal.pcbi.1014426)

**Figure S1**


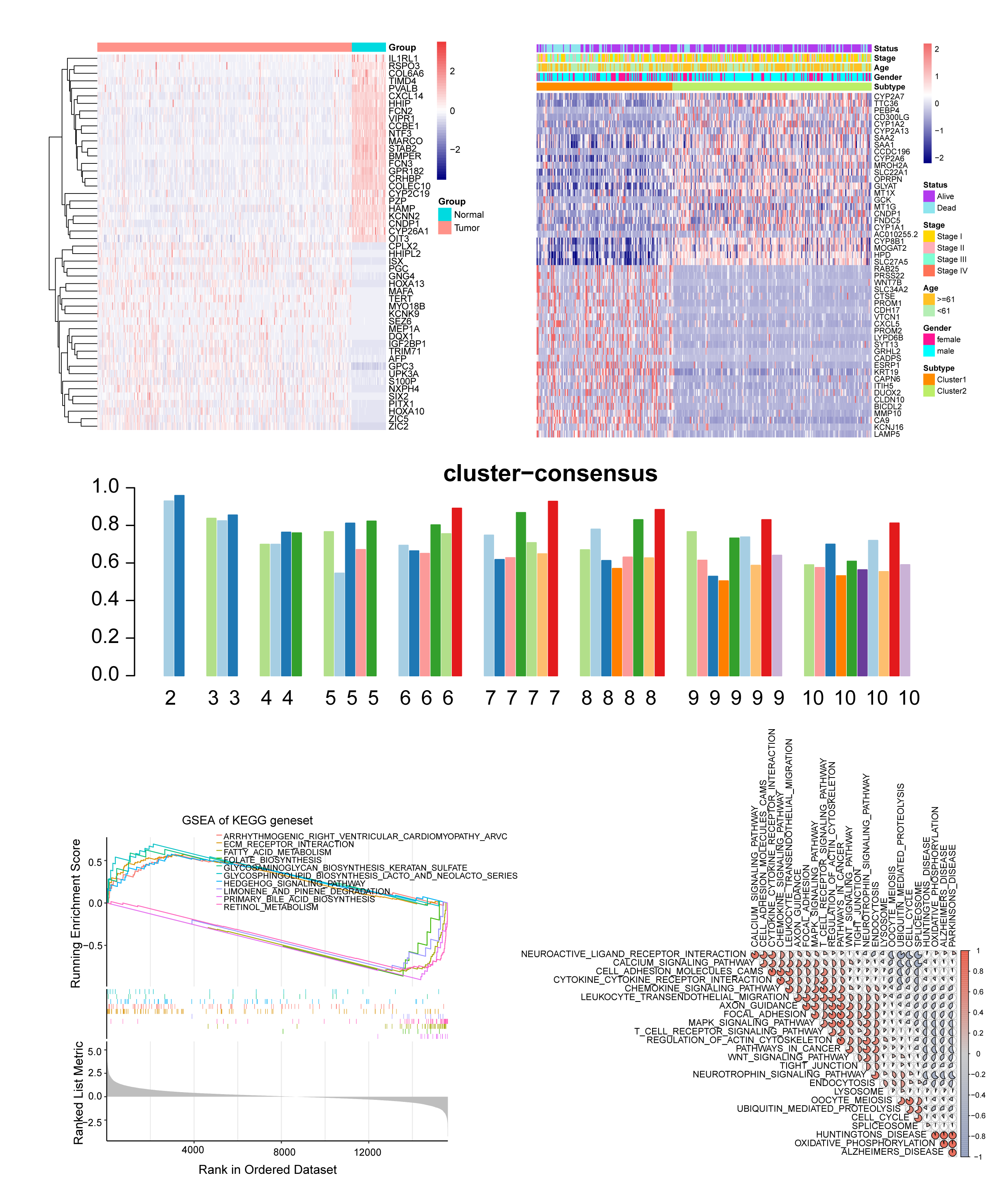

Supplement: S1 Fig — (A) Heatmap of 50 differentially expressed genes, where warm colors represent high expression and cool colors represent low expression. (B) Heatmap of differentially expressed genes among different subgroups, with warm colors representing high expression and cool colors representing low expression. (C) Histogram of consensus values for each K from K = 2 to K = 10 (D) Kyoto Encyclopedia of Genes and Genomes (KEGG) pathway enrichment analysis for genes differentially expressed between Cluster 1 and Cluster 2. (E) Relevance map of molecular pathways. (DOCX) [file pcbi.1014426.s001.docx]

**Figure S2**


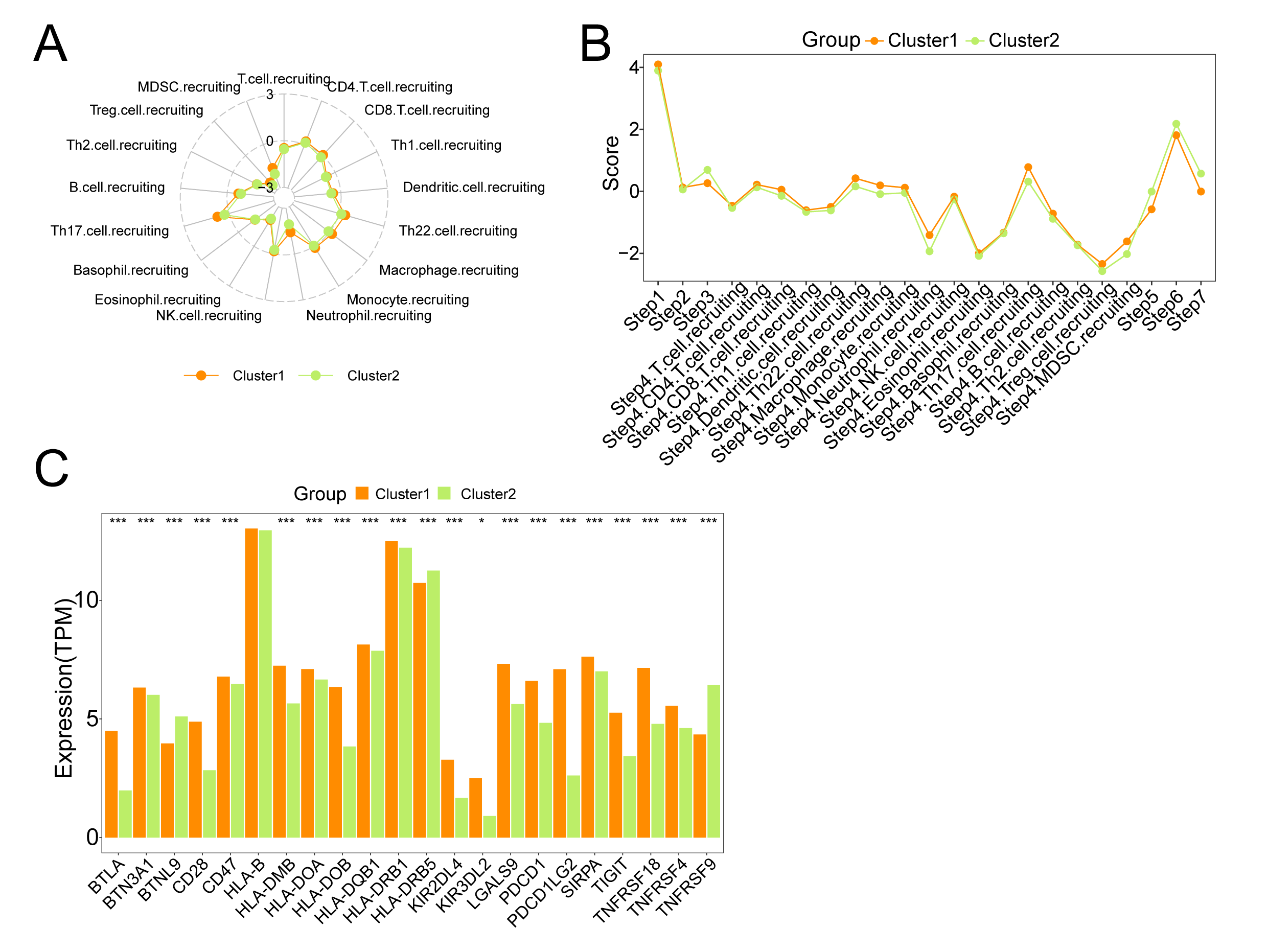

Supplement: S2 Fig — (A) Box plots showing the relative abundance of 12 immune cell types with significant infiltration differences between Cluster 1 and Cluster 2 (CIBERSORT analysis). (B) Immunophenoscore (IPS) comparison between the two subtypes. (C) Comparison of cytolytic activity (CP), effector cells (EC), MHC molecules (MHC), and immune checkpoints (SC) scores between subtypes. (D) Tumor Immune Dysfunction and Exclusion (TIDE) scores, Dysfunction scores, and Exclusion scores between subtypes. (E) Differences in CAF scores between different subtypes. (F) Waterfall plot showing the mutation landscape of top 20 frequently mutated genes in both subtypes. (G) Tumor mutational burden (TMB) comparison between subtypes. *p < 0.05, **p < 0.01, ***p < 0.001. (DOCX) [file pcbi.1014426.s002.docx]

**Figure S3**


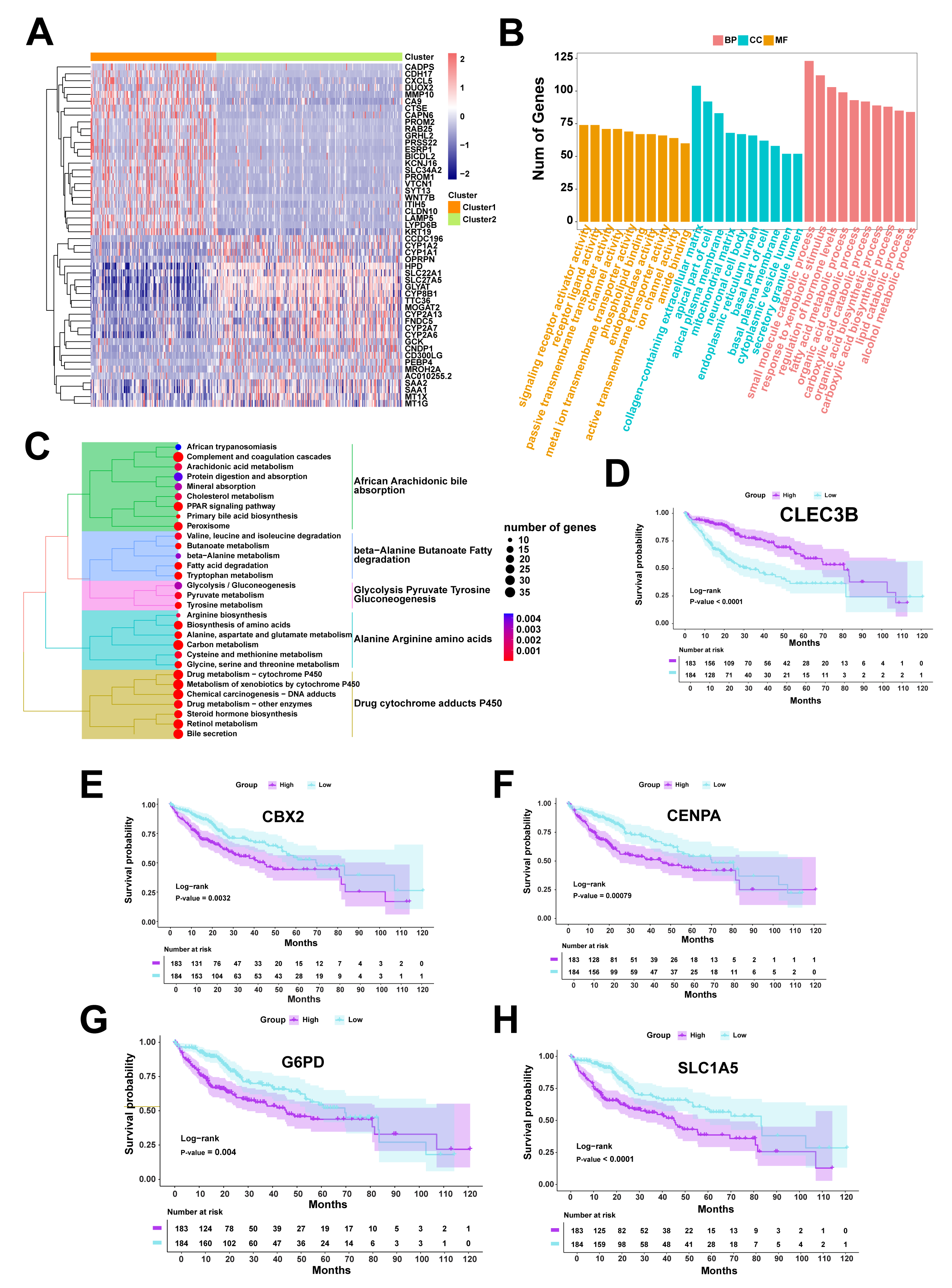

Supplement: S3 Fig — (A) Heatmap of 50 differentially expressed genes, warm colors represent high expression and cool colors represent low expression. (B) GO enrichment plot of subtype-specific differentially expressed genes. Red, blue, and yellow represent BP, CC, and MF terms, respectively. The x-axis represents the enriched pathways, while the y-axis indicates the number of genes enriched in each pathway. (C) KEGG enrichment analysis dendrogram. The size of the circles represents the number of genes, and the color represents the adjusted P-value. Warmer colors correspond to smaller values, while cooler colors indicate larger values. (D-H) KM curve of high and low groups of characteristic genes. (DOCX) [file pcbi.1014426.s003.docx]

**Figure S4**


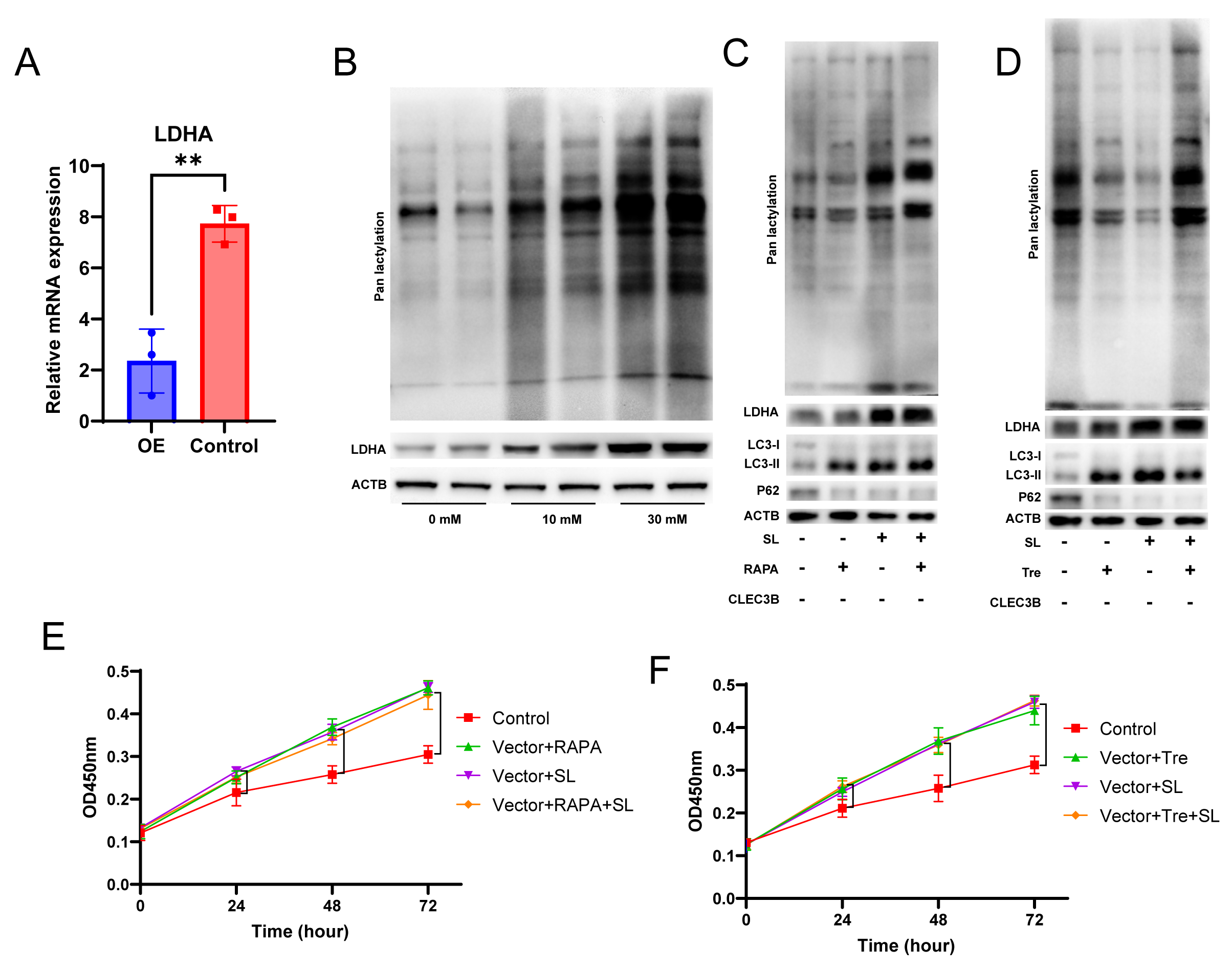

Supplement: S4 Fig — (A) qPCR analysis of LDHA mRNA levels in Huh7 cells treated with Control or OE-LSEC conditioned media. (B) Dose-dependent induction of protein lactylation by sodium lactate in Huh7 cells. (C, D) Western blot analysis of the indicated proteins in Huh7 cells subjected to rescue experiments with conditioned media plus: sodium lactate (SL, 30 mM), rapamycin (RAPA, 100 nM), or trehalose (Tre, 50 mM). (E, F) Corresponding CCK-8 proliferation assays for the rescue experiments. Data are presented as mean ± SD. *p < 0.05, **p < 0.01, ***p < 0.001. (DOCX) [file pcbi.1014426.s004.docx]
