# Supplementary material for: A lactylation- and autophagy-associated prognostic signature reveals LSEC-derived CLEC3B as a novel mediator of hepatocellular carcinoma suppression
Source: PLoS Comput Biol. 2026 Jun 22;22(6):e1014426. doi: 10.1371/journal.pcbi.1014426 (PMC13309010; doi:10.1371/journal.pcbi.1014426)
Supplement: Attachment — Submitted filename: Response to Editors.pdf [file pcbi.1014426.s009.pdf]

Response to Editors
Manuscript ID: PCOMPBIOL-D-25-01959 (previous) / PCOMPBIOL-D-25-02039 (current resubmission) **Title:** A Lactylation- and Autophagy-Associated Prognostic Signature Reveals LSEC-Derived CLEC3B as a Novel Mediator of Hepatocellular Carcinoma Suppression
We sincerely thank the editors for their time and constructive feedback on our previous submission. The primary concern raised was that the logical connection between lactylation and autophagy required stronger justification, as this is an emerging field.
We have thoroughly revised the manuscript to address this core concern. The key modifications are summarized below in a point-by-point response.

Editor's Comment: *This study is of interest in exploring two topics (lactylation and autophagy) together in liver cancer. Despite the traditional view of lactate as a byproduct, lactylation is recently emerging in cancer biology as one of the potential mechanisms underlying the Warburg effect. This field, however, remains largely in its new age. Very rare reports can consolidate on the link between lactylation (especially non-histone) and autophagy in cancer, not only in liver cancer but also in other cancer types. This weakens the rationale stated in the study: "growing evidence suggests an intricate crosstalk between metabolic cues (like lactate) and the autophagic machinery", which is the basis of all subsequent bioinformatics analyses. Given this logical weakness, the study design is questionable, and the authors are suggested to justify/modify the study design.*

Our Response: We sincerely thank the editors for this critical and insightful comment, which we fully agree with. In direct response, we have made substantial revisions on both theoretical and experimental fronts to solidify the logical foundation of our study.

1. We have completely revised the Introduction to more accurately reflect the emerging yet promising nature of this field. We have replaced the overstated claim with a more nuanced discussion and cited several recent, high-impact studies that mechanistically link non-histone lactylation to autophagy regulation in various cancer contexts. These include:

- Lactylation of the transcription factor **TFEB** promoting its nuclear translocation and enhancing autophagic and lysosomal activity.
- Lactylation of **FLCN** suppressing mTORC1 lysosomal localization and promoting TFEB-driven autophagy.
- Lactylation of **FOXO3** sustaining persistent autophagy activation in pancreatic cancer. This provides a much more robust and justified premise for our subsequent bioinformatic and experimental investigation.

2. Most importantly, we have incorporated new experimental data to provide direct functional evidence for the proposed link. We conducted a series of **rescue experiments (now presented as Figure 6I and 6J)**. The results definitively show:

- The anti-tumor effect of CLEC3B is dependent on the suppression of both

lactylation and autophagy.

- Restoring either lactylation (via sodium lactate supplementation) or autophagy (via rapamycin) is sufficient to reverse the anti-proliferative effect of CLEC3B.
- A hierarchical relationship exists, positioning **lactylation upstream of autophagy** in this specific pathway.

These new experiments move beyond correlation and establish a causal link, directly addressing the "logical weakness" and fundamentally strengthening our study design and conclusions.

We believe that these comprehensive revisions have thoroughly addressed the editorial concerns. The revised manuscript now presents a logically sound and mechanistically deeper story. We are deeply grateful for the guidance that led to this significant improvement in our work.
