# Supplementary material for: A lactylation- and autophagy-associated prognostic signature reveals LSEC-derived CLEC3B as a novel mediator of hepatocellular carcinoma suppression
Source: PLoS Comput Biol. 2026 Jun 22;22(6):e1014426. doi: 10.1371/journal.pcbi.1014426 (PMC13309010; doi:10.1371/journal.pcbi.1014426)
Supplement: Attachment — Submitted filename: Response to Reviewers.pdf [file pcbi.1014426.s010.pdf]

Response to Reviewers

We sincerely thank all reviewers for their insightful comments and constructive suggestions, which have significantly improved the quality and clarity of our manuscript. We have carefully addressed each point raised. Our point-by-point responses are detailed below, and all corresponding changes have been incorporated into the revised manuscript.

Reviewer #1

Comment 1: *Lines 350 and 352: The manuscript lists the fifth gene in the prognostic signature as "SLG1A5" (line 349), which is incorrect. The correct gene symbol is SLC1A5 (Solute Carrier Family 1 Member 5).*

Response: We sincerely thank the reviewer for pointing out this critical typographical error. The gene symbol has been corrected to "SLC1A5" throughout the manuscript.

Modifications: The correction has been made in the text, now appearing on lines 450 and 451.

Comment 2: *Line 172: The issue: The manuscript defines the risk score as RiskScore = $h_0(t)$ exp(Coef1 X1 + Coef2 X2 + ... + Coefn Xn). Including the baseline hazard $h_0(t)$ in the risk-score formula is incorrect; the risk score is the linear predictor $\Sigma(Coef_i \times X_i)$ and does not contain the baseline hazard.*

Response: We appreciate the reviewer's meticulous correction. The formula has been revised to the correct form for the risk score calculation.

Modifications: The formula has been amended to `RiskScore = Coef1 X1 + Coef2 X2 + … + Coefn Xn` and is now presented on line 211.

Comment 3: *Lines 210-211: The immunofluorescence method states that sections were fixed in 4 % paraformaldehyde for 12 hours at room temperature. This unusually long fixation at room temperature could compromise tissue integrity; typically, fixation is 10–30 minutes at room temperature or several hours at 4 °C. Please clarify or correct this experimental detail.*

Response: We thank the reviewer for noting this error in the method description. The

stated 12-hour fixation at room temperature was a mistake. The protocol has been corrected to reflect the accurate procedure.

Modifications: The text has been corrected to state "fixed in 4% paraformaldehyde for 20 minutes at room temperature" (line 283).

Comment 4: *Lines 277–280 vs. 329–331 – There is an internal inconsistency in how the clusters are described. At lines 277–280, patients in Cluster 1 are reported to have significantly longer overall survival than those in Cluster 2. However, lines 329–331 refer to Cluster 1 as the "poor-prognosis subtype" and Cluster 2 as the "favorable-prognosis subtype". Use consistent cluster labelling throughout the manuscript and adjust the text and figures accordingly.*

Response: We are grateful to the reviewer for identifying this inconsistency. The survival analysis result was initially misstated. Cluster 1 is indeed the poor-prognosis subtype, and Cluster 2 is the favorable-prognosis subtype. The text has been corrected for consistency.

Modifications: The result description on lines 277-280 has been revised to accurately state that patients in Cluster 2 had significantly longer overall survival than those in Cluster 1 (now lines 378-379). All subsequent references to the clusters have been verified and made consistent.

Comment 5: *Line 365: The text uses informal phrasing ("Anyway, we successfully developed…"). Replace such colloquial language with formal academic wording (e.g., "In summary, we developed…").*

Response: We thank the reviewer for this suggestion to improve the academic tone. The phrasing has been changed accordingly.

Modifications: "Anyway" has been replaced with "In summary" (lines 463-464).

Comment 6: *Line. 429: The rescue experiments use 30 mM sodium lactate without adequate justification. No dose-response assessment is provided, and 30 mM represents the upper physiological limit of lactate accumulation in solid tumors (typically 10–30 mM in the tumor microenvironment, occasionally up to 40–50 mM only in severely hypoxic tumor cores). Such concentrations may be supraphysiological for standard in-vitro culture conditions, where baseline lactate levels are ~1–2 mM. Additionally, no citation is provided for the selection of 30 mM in the context of HCC biology. Please*

include supporting literature or adjust the experimental design accordingly.

Response: We sincerely appreciate the reviewer's insightful comment regarding the justification of the sodium lactate concentration. The reviewer is correct that 30 mM is at the upper physiological limit reported in solid tumors. To address this point comprehensively, we conducted a new dose-response experiment (now included as **Fig. S4B**) to empirically determine an effective yet non-cytotoxic concentration for our model. We treated Huh7 cells with 0, 10, 30, and 50 mM sodium lactate. The results showed that protein lactylation increased in a dose-dependent manner from 0 to 30 mM. However, at 50 mM, significant cell death was observed. Therefore, 30 mM was selected as it induced robust lactylation while maintaining cell viability, representing a balance between effect and toxicity for our *in vitro* rescue experiments.

Modifications: The selection of 30 mM is justified in the text based on the new **Fig. S4B**

Reviewer #2

We thank Reviewer #2 for the thorough and constructive evaluation, which has greatly strengthened the experimental validation and mechanistic discussion in our manuscript.

Scientific Comments:

Comment 1: *Fig. 5F: Specific markers for hepatocytes, NPCs, and LSECs are necessary to demonstrate the purity of each cell population. Additionally, CLEC3B levels should be quantified to support the analysis.*

Response: We agree with the reviewer that validating the purity of isolated primary cells is crucial. As suggested, we have performed additional qPCR experiments to assess the expression of cell-type-specific markers.

For hepatocytes: Alb and Apoa1; For LSECs: Pecam1 and Cd105; For other Non-Parenchymal Cells (NPCs, predominantly immune cells): Cd45.

The results, presented in the new Fig. 5E, confirm the high purity of our isolated cell populations, as each marker was predominantly enriched in its respective cell type.

Furthermore, we have supplemented the Western blot analysis in Fig. 5G with a quantitative densitometry analysis to clearly show the relative protein levels of CLEC3B across the different cell types.

Modifications: The purity validation experiment (qPCR) is now Fig. 5E and described in the Results section (3.5). The quantitative analysis of CLEC3B protein levels is

included in Fig. 5G.

Comment 2: *Immunofluorescence co-staining of Clec3b (green) and Cd105 (red, LSEC marker): Higher-resolution images should be provided, and the relative levels of Clec3b in Cd105-positive and -negative cells should be compared. As strong evidence of CLEC3B localization in LSEC cells, flow cytometry should be performed to measure CLEC3B and LSEC marker expression, which will allow comparison of the fluorescent intensity of CLEC3B between LSEC-positive and LSEC-negative cells.*

Response: We thank the reviewer for these suggestions to strengthen the imaging evidence.

1. Higher-resolution images: We have replaced the immunofluorescence image in Fig. 5H with a higher-resolution version that provides clearer cellular detail.
2. Spatial analysis: In the new high-resolution image, the co-localization of Clec3b (green) and Cd105 (red) is remarkably complete, with nearly 100% overlap observed in the liver sinusoidal structures. Furthermore, we note that the Clec3b signal is strongest in the peripheral liver sinusoids (typical LSEC location) and weaker in central venous cells, reinforcing its LSEC-predominant expression.
3. Flow cytometry: We agree that flow cytometry would be an excellent orthogonal method. However, after an extensive search, we were unable to source a CLEC3B antibody validated for flow cytometry in mice. Despite this limitation, the combined evidence from single-cell RNA-seq (Fig. 5A-D), qPCR (Fig. 5F), Western blot with quantification (Fig. 5G), and high-resolution co-staining (Fig. 5H) provides a consistent and robust multi-modal validation that CLEC3B is predominantly expressed by LSECs.

Modifications:

A higher-resolution image is provided as Fig. 5H.

The observation of the staining pattern is mentioned in the Results (3.5).

The limitation regarding flow cytometry is acknowledged in the response.

Comment 3: *Fig. 6D: The human LSEC cell line used for overexpression should be specified, and the plasmid containing Clec3b, as well as the transduction method, should be described in the methods section.*

Response: We appreciate the reviewer's attention to methodological detail. The requested information has been added to the Methods section.

Modifications: The source and culture conditions of the human LSEC cell line, the

details of the CLEC3B overexpression plasmid, and the lentiviral transduction protocol are now fully described in the "Cell lines and culture" subsection (Methods, lines 256-280).

Comment 4: *Fig. 6E: No ELISA data are shown. This should be addressed.*
Response: We apologize for the oversight. The original figure panel displayed raw OD450 values. We have now processed this data using a standard curve to calculate the actual CLEC3B concentration, and the revised graph is presented accordingly.
Modifications: The ELISA data in the figure (now Fig. 6F, right panel) clearly shows the concentration of secreted CLEC3B in μg/mL.

Comment 5: *It is interesting to observe that both rapamycin and sodium lactate can restore cell proliferation in cells treated with CLEC3B-conditioned media. However, data for cells expressing the vector only (as a control for CLEC3B expression) under the same treatments should be provided. This would help determine whether the effects of rapamycin and sodium lactate are general or specific to CLEC3B-overexpressing cells.*
Response: This is an excellent point. To address it, we performed a new set of control experiments, now presented in Fig. S5B-E. Huh7 cells were treated with conditioned medium from Vector-control LSECs (lacking CLEC3B overexpression). In this context, the addition of rapamycin, trehalose, or sodium lactate effectively promoted protein lactylation, autophagy, and cell proliferation to a similar extent across all treatment groups. This confirms that these agents are general activators of their respective pathways. Therefore, the "rescue" of proliferation shown in the main experiment (Fig. 6J-M) is best interpreted as these general activators overcoming the pathway suppression imposed by CLEC3B, rather than a specific reversal of CLEC3B's molecular action.
Modifications: The new control experiments are presented as Fig. S4C-F. These results and their interpretation are integrated into the Results section (3.6).

Comment 6: *Fig. 6H: The increased LC3II levels could reflect either higher autophagic flux or inhibited autophagic degradation. A further autophagy turnover assay with or without Bafilomycin A1 would clarify this distinction.*
Response: We thank the reviewer for raising this important technical consideration.

While a bafilomycin A1 assay is the gold standard for measuring flux, we employed an alternative and widely accepted approach by examining the levels of p62/SQSTM1, a selective autophagy substrate. If autophagic degradation is functional, activation of autophagy typically leads to p62 degradation (decreased levels). Conversely, accumulation of p62 indicates a block in autophagic flux. In our experiments (now Fig. 6I), the combination of decreased LC3-II and increased p62 upon CLEC3B treatment provides strong evidence for suppressed autophagic activation/degradation, rather than enhanced flux. This logical interpretation is now clarified in the text.
Modifications: The role of p62 as an indicator of autophagic flux is explained in the context of the results (section 3.6).

Comment 7: *Since rapamycin is a weak autophagy inducer, to strengthen the evidence for autophagy involvement (e.g., in Figs. 6I and 6J), other conditions, such as AZD8055, Torin1/2, or starvation, should also be tested.*
Response: We agree that using multiple inducers strengthens the conclusion. In the revised rescue experiments (Figs. 6J, K), we included not only the mTOR-dependent inducer rapamycin but also trehalose, an mTOR-independent autophagy inducer that acts via TFEB activation. The fact that both rapamycin and trehalose produced similar rescue effects (restoring autophagy markers and proliferation) provides stronger and more generalizable evidence for the involvement of the autophagy pathway in mediating CLEC3B's effects.
Modifications: The experiments with trehalose are now included in Figs. 6J, K, L, M and described in the Results (3.6).

Comment 8: *While lactylation is closely related to lactate, the manuscript does not present data correlating sodium lactate levels with lactylation. If not addressed, the claim about lactylation might be overextended. It would be more appropriate to focus on lactate itself rather than lactylation.*
Comment 9: *LDHA is closely related to lactate, but its high level does not necessarily correlate with high lactylation. If the manuscript aims to focus on lactylation, the use of specific antibodies or mass spectrometry data to measure lactylation levels is necessary.*

Response 8 & 9 (Combined): We thank the reviewer for these interrelated comments,

which highlight the need to directly measure lactylation. We have conducted new experiments to directly address the link between lactate, LDHA, and protein lactylation.
1. We performed a dose-response experiment showing that sodium lactate treatment (0-30 mM) increases global protein lactylation in Huh7 cells in a concentration-dependent manner (Fig. S4B).
2. In all key mechanistic figures (Figs. 6I, J, K), we now include Western blot data using a pan-lactylation antibody to directly assess global lactylation levels.
These new data directly demonstrate: (A) that exogenous lactate increases lactylation in our system, and (B) that CLEC3B suppression correlates with reduced levels of both LDHA and protein lactylation. This solidifies our focus on the lactylation modification.
Modifications: The lactate dose-response (lactylation induction) is shown in Fig. S4B. Pan-lactylation blots are included in Figs. 6I, J, K.

Comment 10: *If CLEC3B is indeed important for both lactylation and autophagy, the manuscript only describes their simultaneous response to CLEC3B. However, the potential link between these two processes or whether they are regulated in parallel by CLEC3B is not addressed. This should be discussed, especially considering that the title and abstract suggest such a connection.*
Response: This is a profound and critical question. Our rescue experiments (Figs. 6J-M) provide important insight into this relationship. We observed that restoring lactylation (with sodium lactate) simultaneously restored both lactylation levels and autophagic flux. In contrast, restoring autophagy alone (with rapamycin or trehalose) did not reverse the suppression of lactylation. This unidirectional rescue pattern suggests that, within the mechanism of CLEC3B action, the suppression of lactylation likely lies upstream and contributes to the inhibition of autophagy. We have significantly expanded the Discussion to explicitly present and debate this hierarchical model, exploring potential mechanisms such as lactylation of autophagy-related proteins (e.g., TFEB, ULK1).
Modifications: A substantial paragraph discussing the potential hierarchy between lactylation and autophagy in the context of CLEC3B signaling has been added to the Discussion (lines 589-603).

Technical Comments:
Comment 1: *Most immunofluorescence assays are performed after tissue fixation*

followed by OCT embedding. However, this study uses OCT embedding first, followed by fixation. The reasons for this approach should be clarified.

Response: We apologize for the error in the method description. The standard protocol (fixation followed by OCT embedding) was correctly followed in our experiments. The text has been corrected.

Modifications: The Methods section (lines 283-287) now correctly states that tissues were fixed first and then embedded in OCT compound.

Comment 2: *The CLEC3B antibody from the supplier is intended for Western blot (WB), not for immunofluorescence (IF). This should be addressed.*

Response: We thank the reviewer for their meticulous check. This was an error in the antibody catalog number citation. For immunofluorescence, we used a different, validated antibody. The information has been corrected.

Modifications: The correct antibody details for immunofluorescence (A4387, Abclonal) are now provided in the Methods (line 287).

Comment 3: *The labeling of "tumor-3" and "normal-3" in Fig. 6C should be confirmed to ensure accuracy.*

Response: We are grateful to the reviewer for spotting this labeling error. The labels for the third patient pair in Fig. 6C were indeed reversed and have now been corrected.

Modifications: The labels "tumor-3" and "normal-3" in Fig. 6C have been swapped to their correct positions.

Reviewer #3

We thank Reviewer #3 for the positive assessment of our work and the valuable suggestions for enhancement.

Suggestion 1: *Authors are suggested to include a schematic figure in the manuscript (e.g., in the Discussion or as Fig. 7) that visually summarizes the proposed mechanism: LSEC → secreted CLEC3B → HCC cell → reduced lactylation + inhibited autophagy → suppressed proliferation. This would greatly enhance reader comprehension of the central finding.*

Response: We agree that a graphical summary will greatly improve the clarity of our proposed model. As suggested, we have created a new schematic diagram (Fig. 7)

illustrating the central mechanism: LSEC-derived CLEC3B acts on HCC cells to suppress the LDHA/lactate/lactylation axis and downstream autophagic flux, ultimately inhibiting proliferation.

Modifications: A new graphical abstract figure (Fig. 7) has been added to the manuscript.

Suggestion 2: *Authors should briefly discuss potential upstream regulators or microenvironmental factors that may cause the observed reduction in LSEC abundance and/or CLEC3B secretion in HCC (e.g., hypoxia, inflammation, or angiocrine signaling alterations), even if speculative, to provide better context for why CLEC3B is downregulated in tumors.*

Response: This is an excellent suggestion to provide broader context. We have added a paragraph to the Discussion speculating on potential upstream factors based on the known pathophysiology of LSEC "capillarization" in chronic liver disease and HCC, such as hypoxia (HIF signaling), inflammatory cytokines (e.g., TGF-β, VEGF), and tumor-derived paracrine signals.

Modifications: A new speculative discussion on potential upstream regulators of CLEC3B downregulation has been added (Discussion, lines 604-610).

Suggestion 3: *Authors should clarify and briefly elaborate on the experimental evidence that the anti-proliferative effect of CLEC3B-conditioned medium is specifically mediated by reduced lactylation and autophagy (e.g., mention whether rescue experiments with autophagy activators or lactylation mimics were performed or planned), to strengthen the mechanistic claim.*

Response: We thank the reviewer for this suggestion. We have now more explicitly described and interpreted the rescue experiments in both the Results and Discussion. Specifically, we highlight that the anti-proliferative effect of CLEC3B can be overcome by activating either lactylation (sodium lactate) or autophagy (rapamycin/trehalose), and that the lactylation-activation also restores autophagy, suggesting a hierarchical relationship.

Modifications: The description of the rescue experiments and their mechanistic implication has been strengthened in the Results (section 3.6) and further elaborated in the Discussion (lines 589-603).

Suggestion 4: *Authors are suggested to include one or two recent references (2023–2025) on the emerging role of lactylation in HCC or liver cancer microenvironment (if available), to further strengthen the timeliness and novelty of linking lactylation to autophagy suppression in this context.*

Response: We agree that updating the references enhances the timeliness of the discussion. We have incorporated several recent (2023-2025) key reviews and primary research articles highlighting the pro-tumorigenic roles of lactylation in HCC, including its regulation of metabolism, therapy resistance, and the immune microenvironment.

Modifications: Recent relevant references on lactylation in HCC have been added to the Discussion (lines 573-577).
