# Supplementary material for: A lactylation- and autophagy-associated prognostic signature reveals LSEC-derived CLEC3B as a novel mediator of hepatocellular carcinoma suppression
Source: PLoS Comput Biol. 2026 Jun 22;22(6):e1014426. doi: 10.1371/journal.pcbi.1014426 (PMC13309010; doi:10.1371/journal.pcbi.1014426)
Supplement: Attachment — Submitted filename: Response_to_Reviewers_auresp_2.pdf [file pcbi.1014426.s011.pdf]

Response to Reviewers

We sincerely thank all reviewers for their insightful comments and constructive suggestions, which have significantly improved the quality and clarity of our manuscript. We have carefully addressed each point raised. Our point-by-point responses are detailed below, and all corresponding changes have been incorporated into the revised manuscript.

Reviewer #2

Comments: *The authors have addressed all the comments I raised, and I recommend acceptance.*

Response: We sincerely appreciate the reviewer's positive evaluation and support for our manuscript.

Reviewer #3

Comments: *All previous concerns have been adequately addressed. The revised manuscript is greatly improved.*

Response: We thank the reviewer for this encouraging assessment. We are delighted that the revisions have improved the manuscript.

Reviewer #4

We thank Reviewer #4 for their thorough reading and insightful suggestions. We have carefully addressed each point, primarily through textual enhancements in the Discussion and Methods sections, as recommended.

General Comments

Reviewer Comment 1: *No in vivo validation – all functional work is in vitro with conditioned media. No LSEC-specific knockout/overexpression animal models.*

Response: We fully agree that in vivo validation is a critical next step. We have now

expanded the limitations paragraph in the Discussion (Page 17, Lines 634–636) to more explicitly acknowledge this gap. We also added a new "Outstanding Questions and Future Directions" section (Page 17, Lines 649–652) that specifically proposes the use of LSEC-conditional knockout and overexpression mouse models as a priority for future studies.

Reviewer Comment 2: *Incomplete mechanism:CLEC3B receptor on HCC cells not identified. Signaling pathway between CLEC3B binding to lactylation suppression to autophagy inhibition not elucidated. How does secreted CLEC3B reduce LDHA expression?.*

Response: We appreciate the reviewer for highlighting these mechanistic gaps. As noted above, we have expanded the Discussion to explicitly acknowledge that the CLEC3B receptor remains unknown, the intracellular signaling cascade linking receptor engagement to LDHA suppression has not been delineated, and the direct molecular targets of lactylation within the autophagy machinery await experimental validation (Page 17, Lines 625–634). We have also proposed specific experimental strategies to address these questions, including co-immunoprecipitation coupled with mass spectrometry for receptor identification and CRISPR-Cas9-based receptor screening (Page 19, Lines 640–644). These additions fully address the reviewer's request.

Reviewer Comment 3: *Limited mechanistic depth – While rescue experiments show lactylation is upstream of autophagy, the molecular details are missing.*

Response: We agree. We have now expanded Discussion (Page 17, Lines 625–628) explicitly states that the direct molecular targets of lactylation on autophagy components have not been experimentally validated. We have also proposed lactylome profiling as a future direction to identify specific lactylation sites on autophagy-related

proteins (Page 18, Lines 652–656).

Reviewer Comment 4: *Computational novelty – Standard bioinformatics tools applied competently.*

Response: We thank the reviewer for recognizing our competent application of these tools.

Reviewer Comment 5: *Emerging field caveat – Lactylation-autophagy crosstalk is still a nascent area; even with new citations, the biological foundation remains relatively thin.*

Response: We fully agree. We have added a sentence in the Discussion (Page 16, Lines 607−611) explicitly acknowledging that the lactylation-autophagy crosstalk field is nascent and that our proposed model represents an initial framework requiring refinement as the molecular details are further elucidated.

Reviewer Comment 6: *Fig 6I-J interpretation – The rescue data are correlative. We don't know if these are direct effects or downstream consequences.*

Response: We fully agree with this important point. In the revised Discussion, we have now addressed it directly in the limitations paragraph. Specifically, we have added a sentence (Page 17, Line 628–634) that explicitly acknowledges the correlative nature of the rescue experiments in Figure 6I−J, states that these data do not distinguish between direct molecular targeting and indirect downstream consequences, and proposes future approaches to resolve this mechanistic uncertainty (e.g., broader metabolomic/proteomic profiling and targeted lactylomics). We believe this direct discussion of the limitation in the main text, rather than a brief figure legend edit, provides a substantive response to the reviewer's concern.

Reviewer Comment 7: *Clinical sample size – Only 41 patients from one institution for qPCR/Western validation.*

Response: We acknowledge this limitation. We have expanded the limitations paragraph (Page 17, Line 636-638) to note that future multi-center studies with larger sample sizes are needed to confirm the prognostic and diagnostic utility of serum CLEC3B.

Reviewer Comment 8: *Missing controls – No data showing CLEC3B doesn't affect other metabolic pathways or that the effect is specific to lactylation/autophagy.*

Response: This is a valid point. We have now acknowledged in the revised Discussion (Page 17, Lines 628–634) that we cannot formally exclude the possibility of CLEC3B modulating other pathways, and we propose broader metabolomic or phosphoproteomic profiling as a future direction to assess specificity.

Specific Technical Concerns

Reviewer Comment 1: *Statistical clarification – Ensure all multiple testing corrections are properly described.*

Response: We have revised the Statistical Analysis section (Page 10, Lines 358–361) to explicitly describe the use of the Benjamini-Hochberg procedure for false discovery rate control in all bioinformatics analyses involving multiple hypothesis testing (e.g., DESeq2, clusterProfiler). We have also noted that all specific statistical tests are indicated in the respective figure legends or method sections.

Reviewer Comment 2: *Figure 6I-J legend – Clarify that rescue experiments demonstrate dependence but don't prove direct molecular mechanisms.*

Response: We understand the reviewer's concern and agree that it is important to clarify

the interpretive scope of these rescue experiments. Rather than limiting this clarification to the figure legend—which, by convention, primarily describes the experimental conditions and data shown—we have addressed this point more substantively in the Discussion. Specifically, in the limitations paragraph (Page 17, Line 628–634), we explicitly state that the rescue experiments in Figure 6I–J demonstrate pathway dependence but are correlative in nature, cannot distinguish direct molecular targeting from indirect downstream consequences, and require future targeted lactylomic or mutagenesis studies to resolve the precise molecular mechanisms. We believe that a thorough discussion in the main text provides a more appropriate and complete response to this concern than a brief statement in the figure legend.

Optional Enhancements

Reviewer Comment: *Computational analysis predicting potential receptors from co-expression data; mining published proteomics data for CLEC3B-interacting proteins.*

Response: We appreciate these forward-looking suggestions. While we have not performed these analyses for the current revision due to time constraints, we have incorporated them into the new "Outstanding Questions and Future Directions" section (Page 17, Lines 645–649) as promising approaches for receptor identification, specifically mentioning co-expression network mining and interrogation of published protein-protein interaction databases. We believe this appropriately addresses the reviewer's suggestion within the scope of the current manuscript.
