# Supplementary material for: A lactylation- and autophagy-associated prognostic signature reveals LSEC-derived CLEC3B as a novel mediator of hepatocellular carcinoma suppression
Source: PLoS Comput Biol. 2026 Jun 22;22(6):e1014426. doi: 10.1371/journal.pcbi.1014426 (PMC13309010; doi:10.1371/journal.pcbi.1014426)
Supplement: Peer review history

# Author comment: Author response to Decision Letter 0

**DOI:** 10.1371/journal.pcbi.1014426.r001

9 Oct 2025

Attachment Submitted filename: Response to Editors.pdf

# Peer review: Decision Letter 0

**DOI:** 10.1371/journal.pcbi.1014426.r002

**Authors:** Arne Elofsson, Zhaoyuan Fang

16 Feb 2026

PCOMPBIOL-D-25-02039

A Lactylation- and Autophagy-Associated Prognostic Signature Reveals LSEC-Derived CLEC3B as a Novel Mediator of Hepatocellular Carcinoma Suppression

PLOS Computational Biology

Dear Dr. Liu,

Thank you for submitting your manuscript to PLOS Computational Biology. After careful consideration, we feel that it has merit but does not fully meet PLOS Computational Biology's publication criteria as it currently stands. Therefore, we invite you to submit a revised version of the manuscript that addresses the points raised during the review process.

Please submit your revised manuscript by Apr 18 2026 11:59PM. If you will need more time than this to complete your revisions, please reply to this message or contact the journal office at ploscompbiol@plos.org. When you're ready to submit your revision, log on to https://www.editorialmanager.com/pcompbiol/ and select the 'Submissions Needing Revision' folder to locate your manuscript file.

Please include the following items when submitting your revised manuscript:

* A letter that responds to each point raised by the editor and reviewer(s). You should upload this letter as a separate file labeled 'Response to Reviewers'. This file does not need to include responses to formatting updates and technical items listed in the 'Journal Requirements' section below.

* A marked-up copy of your manuscript that highlights changes made to the original version. You should upload this as a separate file labeled 'Revised Manuscript with Track Changes'.

* An unmarked version of your revised paper without tracked changes. You should upload this as a separate file labeled 'Manuscript'.

If you would like to make changes to your financial disclosure, competing interests statement, or data availability statement, please make these updates within the submission form at the time of resubmission. Guidelines for resubmitting your figure files are available below the reviewer comments at the end of this letter

We look forward to receiving your revised manuscript.

Kind regards,

Zhaoyuan Fang

Academic Editor

PLOS Computational Biology

Arne Elofsson

Section Editor

PLOS Computational Biology

**Journal Requirements:**

If the reviewer comments include a recommendation to cite specific previously published works, please review and evaluate these publications to determine whether they are relevant and should be cited. There is no requirement to cite these works unless the editor has indicated otherwise.

1) Please ensure that the CRediT author contributions listed for every co-author are completed accurately and in full.

At this stage, the following Authors/Authors require contributions: Yufei Han, Jianwei Lan, Liangchen Lei, Binjie Li, Pengpeng Liu, Zhuo Meng, Yinkuan Ning, Youai Song, and Quanyan Liu. Please ensure that the full contributions of each author are acknowledged in the "Add/Edit/Remove Authors" section of our submission form.

The list of CRediT author contributions may be found here: https://journals.plos.org/ploscompbiol/s/authorship#loc-author-contributions

2) Please provide an Author Summary. This should appear in your manuscript between the Abstract (if applicable) and the Introduction, and should be 150-200 words long. The aim should be to make your findings accessible to a wide audience that includes both scientists and non-scientists. Sample summaries can be found on our website under Submission Guidelines:

https://journals.plos.org/ploscompbiol/s/submission-guidelines#loc-parts-of-a-submission

3) Please upload all main figures as separate Figure files in .tif or .eps format. For more information about how to convert and format your figure files please see our guidelines:

https://journals.plos.org/ploscompbiol/s/figures

4) We have noticed that you have uploaded Supporting Information files, but you have not included a list of legends. Please add a full list of legends for your Supporting Information files after the references list.

5) Some material included in your submission may be copyrighted. According to PLOSu2019s copyright policy, authors who use figures or other material (e.g., graphics, clipart, maps) from another author or copyright holder must demonstrate or obtain permission to publish this material under the Creative Commons Attribution 4.0 International (CC BY 4.0) License used by PLOS journals. Please closely review the details of PLOSu2019s copyright requirements here: PLOS Licenses and Copyright. If you need to request permissions from a copyright holder, you may use PLOS's Copyright Content Permission form.

Please respond directly to this email and provide any known details concerning your material's license terms and permissions required for reuse, even if you have not yet obtained copyright permissions or are unsure of your material's copyright compatibility. Once you have responded and addressed all other outstanding technical requirements, you may resubmit your manuscript within Editorial Manager.

Potential Copyright Issues:

i) Figure 6E. Please confirm whether you drew the images / clip-art within the figure panels by hand. If you did not draw the images, please provide (a) a link to the source of the images or icons and their license / terms of use; or (b) written permission from the copyright holder to publish the images or icons under our CC BY 4.0 license. Alternatively, you may replace the images with open source alternatives. See these open source resources you may use to replace images / clip-art:

- https://commons.wikimedia.org

- https://openclipart.org/.

6) In the online submission form, you indicated that  In addition, all data from this study can be obtained from the corresponding author upon reasonable request. All PLOS journals now require all data underlying the findings described in their manuscript to be freely available to other researchers, either

1. In a public repository

2. Within the manuscript itself

3. Uploaded as supplementary information.

This policy applies to all data except where public deposition would breach compliance with the protocol approved by your research ethics board. If your data cannot be made publicly available for ethical or legal reasons (e.g., public availability would compromise patient privacy), please explain your reasons by return email and your exemption request will be escalated to the editor for approval. Your exemption request will be handled independently and will not hold up the peer review process, but will need to be resolved should your manuscript be accepted for publication. One of the Editorial team will then be in touch if there are any issues.

7) Please amend your detailed Financial Disclosure statement. This is published with the article. It must therefore be completed in full sentences and contain the exact wording you wish to be published.

1) State the initials, alongside each funding source, of each author to receive each grant. For example: "This work was supported by the National Institutes of Health (####### to AM; ###### to CJ) and the National Science Foundation (###### to AM)."

2) State what role the funders took in the study. If the funders had no role in your study, please state: "The funders had no role in study design, data collection and analysis, decision to publish, or preparation of the manuscript."

3) If any authors received a salary from any of your funders, please state which authors and which funders..

If you did not receive any funding for this study, please simply state: u201cThe authors received no specific funding for this work.u201d

8)  Please ensure that the funders and grant numbers match between the Financial Disclosure field and the Funding Information tab in your submission form. Note that the funders must be provided in the same order in both places as well.

**Reviewers' comments:**

Reviewer's Responses to Questions

**Comments to the Authors:**

**Please note here if the review is uploaded as an attachment.**

Reviewer #1: 1. Lines 350 and 352: The manuscript lists the fifth gene in the prognostic signature as "SLG1A5" (line 349), which is incorrect. The correct gene symbol is SLC1A5 (Solute Carrier Family 1 Member 5).

2. Line 172: The issue: The manuscript defines the risk score as RiskScore = h₀(t) * exp(Coef1 * X1 + Coef2 * X2 + … + Coefn * Xn). Including the baseline hazard h₀(t) in the risk‑score formula is incorrect; the risk score is the linear predictor Σ(Coefᵢ × Xᵢ) and does not contain the baseline hazard.

3. Lines 210-211: The immunofluorescence method states that sections were fixed in 4 % paraformaldehyde for 12 hours at room temperature. This unusually long fixation at room temperature could compromise tissue integrity; typically, fixation is 10–30 minutes at room temperature or several hours at 4 °C. Please clarify or correct this experimental detail.

4. Lines 277–280 vs. 329–331 – There is an internal inconsistency in how the clusters are described. At lines 277–280, patients in Cluster 1 are reported to have significantly longer overall survival than those in Cluster 2. However, lines 329–331 refer to Cluster 1 as the “poor‑prognosis subtype” and Cluster 2 as the “favorable‑prognosis subtype”. Use consistent cluster labelling throughout the manuscript and adjust the text and figures accordingly.

5. Line 365:Tthe text uses informal phrasing (“Anyway, we successfully developed…”). Replace such colloquial language with formal academic wording (e.g., “In summary, we developed…”).

6. Line. 429: The rescue experiments use 30 mM sodium lactate without adequate justification. No dose-response assessment is provided, and 30 mM represents the upper physiological limit of lactate accumulation in solid tumors (typically 10–30 mM in the tumor microenvironment, occasionally up to 40–50 mM only in severely hypoxic tumor cores). Such concentrations may be supraphysiological for standard in-vitro culture conditions, where baseline lactate levels are ~1–2 mM. Additionally, no citation is provided for the selection of 30 mM in the context of HCC biology. Please include supporting literature or adjust the experimental design accordingly.

Supporting references: He Y, Song T, Ning J, Wang Z, Yin Z, Jiang P, Yuan Q, Yu W, Cheng F. Lactylation in cancer: Mechanisms in tumour biology and therapeutic potentials. Clin Transl Med. 2024 Nov;14(11):e70070. doi: 10.1002/ctm2.70070. PMID: 39456119; PMCID: PMC11511673.

Reviewer #2: Studying the crosstalk between lactylation and autophagy is an important step toward understanding the underlying mechanisms of autophagy regulation and the function of lactylation. To clarify the molecular mechanisms linking the lactylation-autophagy interaction to patient survival, the subtype with the worst prognosis (Cluster 1) was compared with the subtype with the best prognosis (Cluster 2). Differentially expressed genes (DEGs) between these two subtypes were identified. Patients in Cluster 1 exhibited significantly longer overall survival (OS) and different immune infiltration profiles compared to those in Cluster 2. Based on these findings, a 5-gene prognostic signature was constructed and validated, based on HCC molecular subtypes. This analysis revealed that high expression of CLEC3B was associated with a favorable prognosis, which is promising. However, when moving to the experimental validation (from the section Single-Cell and Experimental Validation of CLEC3B as a Liver Sinusoidal Endothelial Cell-Specific Gene to the conclusion), more evidence is needed to substantiate the findings.

Scientific Comments:

1. Fig. 5F: Specific markers for hepatocytes, NPCs, and LSECs are necessary to demonstrate the purity of each cell population. Additionally, CLEC3B levels should be quantified to support the analysis.

2. Immunofluorescence co-staining of Clec3b (green) and Cd105 (red, LSEC marker): Higher-resolution images should be provided, and the relative levels of Clec3b in Cd105-positive and -negative cells should be compared. As strong evidence of CLEC3B localization in LSEC cells, flow cytometry should be performed to measure CLEC3B and LSEC marker expression, which will allow comparison of the fluorescent intensity of CLEC3B between LSEC-positive and LSEC-negative cells.

3. Fig. 6D: The human LSEC cell line used for overexpression should be specified, and the plasmid containing Clec3b, as well as the transduction method, should be described in the methods section.

4. Fig. 6E: No ELISA data are shown. This should be addressed.

5. It is interesting to observe that both rapamycin and sodium lactate can restore cell proliferation in cells treated with CLEC3B-conditioned media. However, data for cells expressing the vector only (as a control for CLEC3B expression) under the same treatments should be provided. This would help determine whether the effects of rapamycin and sodium lactate are general or specific to CLEC3B-overexpressing cells.

6. Fig. 6H: The increased LC3II levels could reflect either higher autophagic flux or inhibited autophagic degradation. A further autophagy turnover assay with or without Bafilomycin A1 would clarify this distinction.

7. Since rapamycin is a weak autophagy inducer, to strengthen the evidence for autophagy involvement (e.g., in Figs. 6I and 6J), other conditions, such as AZD8055, Torin1/2, or starvation, should also be tested.

8. While lactylation is closely related to lactate, the manuscript does not present data correlating sodium lactate levels with lactylation. If not addressed, the claim about lactylation might be overextended. It would be more appropriate to focus on lactate itself rather than lactylation.

9. LDHA is closely related to lactate, but its high level does not necessarily correlate with high lactylation. If the manuscript aims to focus on lactylation, the use of specific antibodies or mass spectrometry data to measure lactylation levels is necessary.

10. If CLEC3B is indeed important for both lactylation and autophagy, the manuscript only describes their simultaneous response to CLEC3B. However, the potential link between these two processes or whether they are regulated in parallel by CLEC3B is not addressed. This should be discussed, especially considering that the title and abstract suggest such a connection.

Technical Comments:

1. Most immunofluorescence assays are performed after tissue fixation followed by OCT embedding. However, this study uses OCT embedding first, followed by fixation. The reasons for this approach should be clarified.

2. The CLEC3B antibody from the supplier is intended for Western blot (WB), not for immunofluorescence (IF). This should be addressed.

3. The labeling of "tumor-3" and "normal-3" in Fig. 6C should be confirmed to ensure accuracy.

Reviewer #3: This study integrates multi-omics data to identify two HCC molecular subtypes with distinct prognosis and develops a lactylation-autophagy-related prognostic signature centered on CLEC3B. Single-cell RNA-seq and experimental validation reveal that CLEC3B is primarily secreted by LSECs, markedly downregulated in HCC. Conditioned medium from CLEC3B-overexpressing LSECs inhibits HCC cell proliferation by simultaneously suppressing autophagy and reducing lactylation levels, positioning LSEC-derived CLEC3B as a novel tumor suppressor bridging these pathways with strong prognostic and therapeutic potential.

Overall, this is a high-quality, well-executed study with clear novelty, strong methodology, and significant translational potential. I recommend acceptance after minor revisions.

Suggestions:

1. Authors are suggested to include a schematic figure in the manuscript (e.g., in the Discussion or as Fig. 7) that visually summarizes the proposed mechanism: LSEC → secreted CLEC3B → HCC cell → reduced lactylation + inhibited autophagy → suppressed proliferation. This would greatly enhance reader comprehension of the central finding.

2. Authors should briefly discuss potential upstream regulators or microenvironmental factors that may cause the observed reduction in LSEC abundance and/or CLEC3B secretion in HCC (e.g., hypoxia, inflammation, or angiocrine signaling alterations), even if speculative, to provide better context for why CLEC3B is downregulated in tumors.

3. Authors should clarify and briefly elaborate on the experimental evidence that the anti-proliferative effect of CLEC3B-conditioned medium is specifically mediated by reduced lactylation and autophagy (e.g., mention whether rescue experiments with autophagy activators or lactylation mimics were performed or planned), to strengthen the mechanistic claim.

4. Authors are suggested to include one or two recent references (2023–2025) on the emerging role of lactylation in HCC or liver cancer microenvironment (if available), to further strengthen the timeliness and novelty of linking lactylation to autophagy suppression in this context.

**********

**Have the authors made all data and (if applicable) computational code underlying the findings in their manuscript fully available?**

The PLOS Data policy requires authors to make all data and code underlying the findings described in their manuscript fully available without restriction, with rare exception (please refer to the Data Availability Statement in the manuscript PDF file). The data and code should be provided as part of the manuscript or its supporting information, or deposited to a public repository. For example, in addition to summary statistics, the data points behind means, medians and variance measures should be available. If there are restrictions on publicly sharing data or code —e.g. participant privacy or use of data from a third party—those must be specified.

Reviewer #1: **No:** I did not find the codes (R,...etc codes attached)

Reviewer #2: Yes

Reviewer #3: Yes

**********

PLOS authors have the option to publish the peer review history of their article (what does this mean?). If published, this will include your full peer review and any attached files.

If you choose “no”, your identity will remain anonymous but your review may still be made public.

**Do you want your identity to be public for this peer review?**  For information about this choice, including consent withdrawal, please see our Privacy Policy.

Reviewer #1: No

Reviewer #2: No

Reviewer #3: **Yes:** Shantanu Gupta

**Figure resubmission:**

While revising your submission, we strongly recommend that you use PLOS’s NAAS tool (https://ngplosjournals.pagemajik.ai/artanalysis) to test your figure files. NAAS can convert your figure files to the TIFF file type and meet basic requirements (such as print size, resolution), or provide you with a report on issues that do not meet our requirements and that NAAS cannot fix.

After uploading your figures to PLOS’s NAAS tool - https://ngplosjournals.pagemajik.ai/artanalysis, NAAS will process the files provided and display the results in the "Uploaded Files" section of the page as the processing is complete. If the uploaded figures meet our requirements (or NAAS is able to fix the files to meet our requirements), the figure will be marked as "fixed" above. If NAAS is unable to fix the files, a red "failed" label will appear above. When NAAS has confirmed that the figure files meet our requirements, please download the file via the download option, and include these NAAS processed figure files when submitting your revised manuscript.

**Reproducibility:**

To enhance the reproducibility of your results, we recommend that authors of applicable studies deposit laboratory protocols in protocols.io, where a protocol can be assigned its own identifier (DOI) such that it can be cited independently in the future. Additionally, PLOS ONE offers an option to publish peer-reviewed clinical study protocols. Read more information on sharing protocols at https://plos.org/protocols?utm_medium=editorial-email&utm_source=authorletters&utm_campaign=protocols

# Author comment: Author response to Decision Letter 1

**DOI:** 10.1371/journal.pcbi.1014426.r003

9 Mar 2026

Attachment Submitted filename: Response to Reviewers.pdf

# Peer review: Decision Letter 1

**DOI:** 10.1371/journal.pcbi.1014426.r004

**Authors:** Arne Elofsson, Zhaoyuan Fang, Arne Elofsson, Zhaoyuan Fang

22 May 2026

PCOMPBIOL-D-25-02039R1

A Lactylation- and Autophagy-Associated Prognostic Signature Reveals LSEC-Derived CLEC3B as a Novel Mediator of Hepatocellular Carcinoma Suppression

PLOS Computational Biology

Dear Dr. Liu,

Thank you for submitting your revised manuscript to PLOS Computational Biology. After careful peer review and consideration, we feel that it has merit though not fully meet PLOS Computational Biology's publication criteria as it currently stands. Therefore, we invite you to submit a further revised version of the manuscript that addresses the points raised during the review process.

Please submit your revised manuscript by Jul 22 2026 11:59PM. If you will need more time than this to complete your revisions, please reply to this message or contact the journal office at ploscompbiol@plos.org. When you're ready to submit your revision, log on to https://www.editorialmanager.com/pcompbiol/ and select the 'Submissions Needing Revision' folder to locate your manuscript file.

Please include the following items when submitting your revised manuscript:

* A letter that responds to each point raised by the editor and reviewer(s). You should upload this letter as a separate file labeled 'Response to Reviewers'. This file does not need to include responses to formatting updates and technical items listed in the 'Journal Requirements' section below.

* A marked-up copy of your manuscript that highlights changes made to the original version. You should upload this as a separate file labeled 'Revised Manuscript with Track Changes'.

* An unmarked version of your revised paper without tracked changes. You should upload this as a separate file labeled 'Manuscript'.

If you would like to make changes to your financial disclosure, competing interests statement, or data availability statement, please make these updates within the submission form at the time of resubmission. Guidelines for resubmitting your figure files are available below the reviewer comments at the end of this letter.

As the corresponding author, your ORCID iD is verified in the submission system and will appear in the published article. PLOS supports the use of ORCID, and we encourage all coauthors to register for an ORCID iD and use it as well. Please encourage your coauthors to verify their ORCID iD within the submission system before final acceptance, as unverified ORCID iDs will not appear in the published article. Only the individual author can complete the verification step; PLOS staff cannot verify ORCID iDs on behalf of authors.

We look forward to receiving your revised manuscript.

Kind regards,

Zhaoyuan Fang

Academic Editor

PLOS Computational Biology

Arne Elofsson

Section Editor

PLOS Computational Biology

**Reviewers' comments:**

Reviewer's Responses to Questions

**Comments to the Authors:**

**Please note here if the review is uploaded as an attachment.**

Reviewer #2: The authors have addressed all the comments I raised, and I recommend acceptance.

Reviewer #3: All previous concerns have been adequately addressed. The revised manuscript is greatly improved.

Reviewer #4: No in vivo validation: Authors acknowledge this - all functional work is in vitro with conditioned media. No LSEC-specific knockout/overexpression animal models

Incomplete mechanism:

CLEC3B receptor on HCC cells not identified

Signaling pathway between CLEC3B binding to lactylation suppression to autophagy inhibition not elucidated

How does secreted CLEC3B reduce LDHA expression?

Limited mechanistic depth: While rescue experiments show lactylation is upstream of autophagy, the molecular details are missing

Computational novelty: Standard bioinformatics tools applied competently

Emerging field caveat: Lactylation-autophagy crosstalk is still a nascent area; even with new citations, the biological foundation remains relatively thin

Specific Technical Concerns:

Fig 6I-J interpretation: The rescue data are correlative. CLEC3B reduces LDHA and LC3-II/LC3-I, but we don't know if these are direct effects or downstream consequences

Clinical sample size: Only 41 patients from one institution for qPCR/Western validation

Missing controls: No data showing CLEC3B doesn't affect other metabolic pathways or that the effect is specific to lactylation/autophagy.

I would recommend that the author add these minor enhancements:

Discussion expansion: More explicit acknowledgment that the mechanism is incomplete and the receptor/signaling pathway remain unknown

Future directions: Specific experiments needed (receptor identification, in vivo validation, detailed signaling pathway)

Statistical clarification: Ensure all multiple testing corrections are properly described so that if anyone takes this work can reproduce the same results

Figure 6I-J legend: Clarify that rescue experiments demonstrate dependence on these pathways but don't prove direct molecular mechanisms

Optional enhancements (if time permits):

Computational analysis predicting potential receptors from co-expression data

Mining published proteomics data for CLEC3B-interacting proteins

**********

**Have the authors made all data and (if applicable) computational code underlying the findings in their manuscript fully available?**

The PLOS Data policy requires authors to make all data and code underlying the findings described in their manuscript fully available without restriction, with rare exception (please refer to the Data Availability Statement in the manuscript PDF file). The data and code should be provided as part of the manuscript or its supporting information, or deposited to a public repository. For example, in addition to summary statistics, the data points behind means, medians and variance measures should be available. If there are restrictions on publicly sharing data or code —e.g. participant privacy or use of data from a third party—those must be specified.

Reviewer #2: Yes

Reviewer #3: Yes

Reviewer #4: Yes

**********

PLOS authors have the option to publish the peer review history of their article (what does this mean?). If published, this will include your full peer review and any attached files.

If you choose “no”, your identity will remain anonymous but your review may still be made public.

**Do you want your identity to be public for this peer review?**  For information about this choice, including consent withdrawal, please see our Privacy Policy.

Reviewer #2: No

Reviewer #3: **Yes:** Shantanu Gupta

Reviewer #4: No

[NOTE: If reviewer comments were submitted as an attachment file, they will be attached to this email and accessible via the submission site. Please log into your account, locate the manuscript record, and check for the action link "View Attachments". If this link does not appear, there are no attachment files.]

**Figure resubmission:**

-->While revising your submission, we strongly recommend that you use PLOS’s NAAS tool (https://ngplosjournals.pagemajik.ai/artanalysis) to test your figure files. NAAS can convert your figure files to the TIFF file type and meet basic requirements (such as print size, resolution), or provide you with a report on issues that do not meet our requirements and that NAAS cannot fix.

After uploading your figures to PLOS’s NAAS tool - https://ngplosjournals.pagemajik.ai/artanalysis, NAAS will process the files provided and display the results in the "Uploaded Files" section of the page as the processing is complete. If the uploaded figures meet our requirements (or NAAS is able to fix the files to meet our requirements), the figure will be marked as "fixed" above. If NAAS is unable to fix the files, a red "failed" label will appear above. When NAAS has confirmed that the figure files meet our requirements, please download the file via the download option, and include these NAAS processed figure files when submitting your revised manuscript.

**Reproducibility:**

To enhance the reproducibility of your results, we recommend that authors of applicable studies deposit laboratory protocols in protocols.io, where a protocol can be assigned its own identifier (DOI) such that it can be cited independently in the future. Additionally, PLOS ONE offers an option to publish peer-reviewed clinical study protocols. Read more information on sharing protocols at https://plos.org/protocols?utm_medium=editorial-email&utm_source=authorletters&utm_campaign=protocols

# Author comment: Author response to Decision Letter 2

**DOI:** 10.1371/journal.pcbi.1014426.r005

27 May 2026

Attachment Submitted filename: Response_to_Reviewers_auresp_2.pdf

# Peer review: Decision Letter 2

**DOI:** 10.1371/journal.pcbi.1014426.r006

**Authors:** Arne Elofsson, Zhaoyuan Fang, Arne Elofsson, Zhaoyuan Fang, Arne Elofsson, Zhaoyuan Fang

9 Jun 2026

Dear Dr Liu,

We are pleased to inform you that your manuscript 'A Lactylation- and Autophagy-Associated Prognostic Signature Reveals LSEC-Derived CLEC3B as a Novel Mediator of Hepatocellular Carcinoma Suppression' has been provisionally accepted for publication in PLOS Computational Biology.

Before your manuscript can be formally accepted you will need to complete some formatting changes, which you will receive in a follow up email. A member of our team will be in touch with a set of requests.

Please note that your manuscript will not be scheduled for publication until you have made the required changes, so a swift response is appreciated.

IMPORTANT: The editorial review process is now complete. PLOS will only permit corrections to spelling, formatting or significant scientific errors from this point onwards. Requests for major changes, or any which affect the scientific understanding of your work, will cause delays to the publication date of your manuscript.

Should you, your institution's press office or the journal office choose to press release your paper, you will automatically be opted out of early publication. We ask that you notify us now if you or your institution is planning to press release the article. All press must be co-ordinated with PLOS.

Thank you again for supporting Open Access publishing; we are looking forward to publishing your work in PLOS Computational Biology.

Best regards,

Zhaoyuan Fang

Academic Editor

PLOS Computational Biology

Arne Elofsson

Section Editor

PLOS Computational Biology

***********************************************************

Reviewer's Responses to Questions

**Comments to the Authors:**

**Please note here if the review is uploaded as an attachment.**

Reviewer #4: I have carefully read the revised manuscript and the authors' responses to all reviewer comments. The revisions are satisfactory and adequately address the concerns raised in the previous round.

Specifically, the authors have:

Explicitly acknowledged the absence of in vivo validation and proposed concrete future directions including LSEC-conditional knockout models

Clearly stated the unknown identity of the CLEC3B receptor and outlined specific strategies (co-IP/mass spectrometry, CRISPR-Cas9 screening) to address this

Appropriately qualified the rescue experiments in Figure 6I–J as correlative in nature within the Discussion

Added acknowledgment that the lactylation-autophagy crosstalk field is nascent and that their model is an initial framework

Clarified multiple testing correction procedures in the Statistical Analysis section

Noted the limitation of the small single-institution clinical cohort and the need for future multi-center validation

The manuscript presents a coherent and novel biological axis LSEC-derived CLEC3B suppressing HCC via concurrent inhibition of lactylation and autophagy supported by multi-omics analysis, single-cell validation, and functional experiments. The limitations are transparently disclosed rather than obscured.

Congratulations!

**********

**Have the authors made all data and (if applicable) computational code underlying the findings in their manuscript fully available?**

The PLOS Data policy requires authors to make all data and code underlying the findings described in their manuscript fully available without restriction, with rare exception (please refer to the Data Availability Statement in the manuscript PDF file). The data and code should be provided as part of the manuscript or its supporting information, or deposited to a public repository. For example, in addition to summary statistics, the data points behind means, medians and variance measures should be available. If there are restrictions on publicly sharing data or code —e.g. participant privacy or use of data from a third party—those must be specified.

Reviewer #4: Yes

**********

PLOS authors have the option to publish the peer review history of their article (what does this mean?). If published, this will include your full peer review and any attached files.

If you choose “no”, your identity will remain anonymous but your review may still be made public.

**Do you want your identity to be public for this peer review?**  For information about this choice, including consent withdrawal, please see our Privacy Policy.

Reviewer #4: No

# Editor report: Acceptance letter

**DOI:** 10.1371/journal.pcbi.1014426.r007

**Authors:** Arne Elofsson, Zhaoyuan Fang, Arne Elofsson, Zhaoyuan Fang, Arne Elofsson, Zhaoyuan Fang

PCOMPBIOL-D-25-02039R2

A Lactylation- and Autophagy-Associated Prognostic Signature Reveals LSEC-Derived CLEC3B as a Novel Mediator of Hepatocellular Carcinoma Suppression

Dear Dr Liu,

I am pleased to inform you that your manuscript has been formally accepted for publication in PLOS Computational Biology. Your manuscript is now with our production department and you will be notified of the publication date in due course.

The corresponding author will soon be receiving a typeset proof for review, to ensure errors have not been introduced during production. Please review the PDF proof of your manuscript carefully, as this is the last chance to correct any errors. Please note that major changes, or those which affect the scientific understanding of the work, will likely cause delays to the publication date of your manuscript.

Soon after your final files are uploaded, unless you have opted out, the early version of your manuscript will be published online. The date of the early version will be your article's publication date. The final article will be published to the same URL, and all versions of the paper will be accessible to readers.

For Research, Software, and Methods articles, you will receive an invoice from PLOS for your publication fee after your manuscript has reached the completed accept phase. If you receive an email requesting payment before acceptance or for any other service, this may be a phishing scheme. Learn how to identify phishing emails and protect your accounts at https://explore.plos.org/phishing.

Thank you again for supporting PLOS Computational Biology and open-access publishing. We are looking forward to publishing your work!

With kind regards,

Sharmila Kamatchi

PLOS Computational Biology | Carlyle House, Carlyle Road, Cambridge CB4 3DN | United Kingdom ploscompbiol@plos.org | Phone +44 (0) 1223-442824 | ploscompbiol.org | @PLOSCompBiol
